# Supplementary material for: Pericytes are organ-specific regulators of tissue morphogenesis
Source: Nat Commun. 2026 May 12;17:4229. doi: 10.1038/s41467-026-71643-1 (PMC13168628; doi:10.1038/s41467-026-71643-1)
Supplement: Supplementary file 1 — Supplementary Information [file 41467_2026_71643_MOESM1_ESM.pdf]

Pericytes are organ-specific regulators of tissue morphogenesis

Seyed Javad Rasouli[1], Kai Kruse[2], Rodrigo Diéguez-Hurtado[1], Parisa Ghanbari[3], Anusha Aravamudhan[1], Mara-Elena Pitulescu[1,4], and Ralf H. Adams[1#]

[1]Max Planck Institute for Molecular Biomedicine, Department of Tissue Morphogenesis, D-48149 Münster, Germany.

[2]Max Planck Institute for Molecular Biomedicine, Bioinformatics Service Unit, D-48149, Münster, Germany.

[3]Max Planck Institute for Molecular Biomedicine, Department of Vascular Cell Biology, D-48149, Münster, Germany.

[4]Max Planck Institute for Molecular Biomedicine, Vascular Patterning Dynamics Group, D-48149, Münster, Germany.

[#]Author for correspondence:

Ralf H. Adams
Department of Tissue Morphogenesis
Max-Planck-Institute for Molecular Biomedicine and University of Münster
D–48149 Münster, Germany
ralf.adams@mpi-muenster.mpg.de
Phone: +49 251 70365 410; Fax: +49 251 70365 499

Keywords: pericytes, mural cells, lung, brain, angiocrine signaling, HGF, BDNF, Nodal

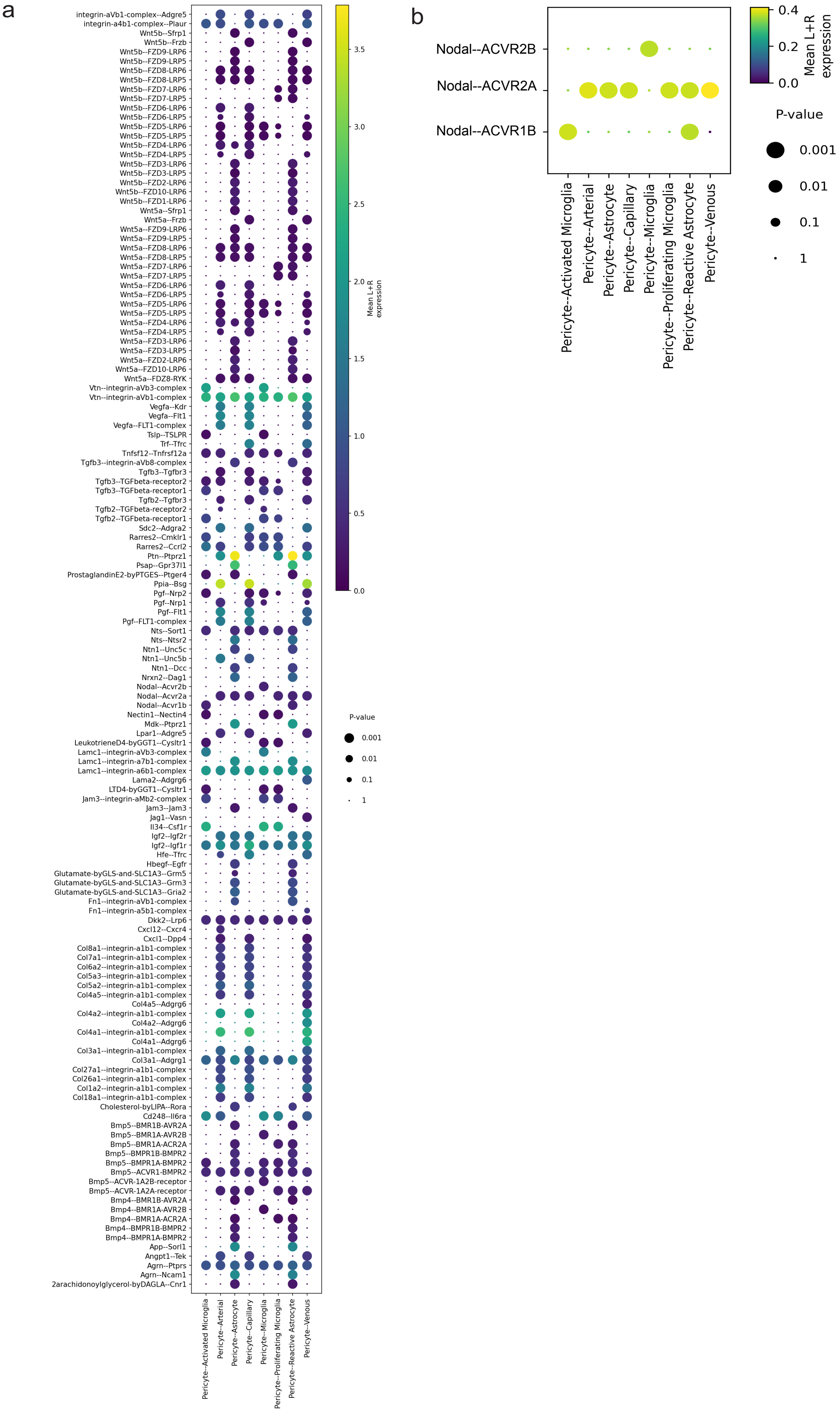

a
integrin-aVb1-complex--Adgre5
integrin-a4b1-complex--Plaur
Wnt5b--Sfrp1
Wnt5b--Frzb
Wnt5b--FZD9-LRP6
Wnt5b--FZD9-LRP5
Wnt5b--FZD8-LRP6
Wnt5b--FZD8-LRP5
Wnt5b--FZD7-LRP6
Wnt5b--FZD7-LRP5
Wnt5b--FZD6-LRP6
Wnt5b--FZD6-LRP5
Wnt5b--FZD5-LRP6
Wnt5b--FZD5-LRP5
Wnt5b--FZD4-LRP6
Wnt5b--FZD4-LRP5
Wnt5b--FZD3-LRP6
Wnt5b--FZD3-LRP5
Wnt5b--FZD2-LRP6
Wnt5b--FZD10-LRP6
Wnt5b--FZD1-LRP6
Wnt5a--Sfrp1
Wnt5a--Frzb
Wnt5a--FZD9-LRP6
Wnt5a--FZD9-LRP5
Wnt5a--FZD8-LRP6
Wnt5a--FZD8-LRP5
Wnt5a--FZD7-LRP6
Wnt5a--FZD7-LRP5
Wnt5a--FZD6-LRP6
Wnt5a--FZD6-LRP5
Wnt5a--FZD5-LRP6
Wnt5a--FZD5-LRP5
Wnt5a--FZD4-LRP6
Wnt5a--FZD4-LRP5
Wnt5a--FZD3-LRP6
Wnt5a--FZD3-LRP5
Wnt5a--FZD2-LRP6
Wnt5a--FZD10-LRP6
Wnt5a--FDZ8-RYK
Vtn--integrin-aVb3-complex
Vtn--integrin-aVb1-complex
Vegfa--Kdr
Vegfa--Flt1
Vegfa--FLT1-complex
Tslp--TSLPR
Trf--Tfrc
Tnfsf12--Tnfrsf12a
Tgfb3--integrin-aVb8-complex
Tgfb3--Tgfbr3
Tgfb3--TGFbeta-receptor2
Tgfb3--TGFbeta-receptor1
Tgfb2--Tgfbr3
Tgfb2--TGFbeta-receptor2
Tgfb2--TGFbeta-receptor1
Sdc2--Adgra2
Rarres2--Cmklr1
Rarres2--Ccrl2
Ptn--Ptprz1
Psap--Gpr37l1
ProstaglandinE2-byPTGES--Ptger4
Ppia--Bsg
Pgf--Nrp2
Pgf--Nrp1
Pgf--Flt1
Pgf--FLT1-complex
Nts--Sort1
Nts--Ntsr2
Ntn1--Unc5c
Ntn1--Unc5b
Ntn1--Dcc
Nrxn2--Dag1
Nodal--Acvr2b
Nodal--Acvr2a
Nodal--Acvr1b
Nectin1--Nectin4
Mdk--Ptprz1
Lpar1--Adgre5
LeukotrieneD4-byGGT1--Cysltr1
Lamc1--integrin-aVb3-complex
Lamc1--integrin-a7b1-complex
Lamc1--integrin-a6b1-complex
Lama2--Adgrg6
LTD4-byGGT1--Cysltr1
Jam3--integrin-aMb2-complex
Jam3--Jam3
Jag1--Vasn
Il34--Csf1r
Igf2--Igf2r
Igf2--Igf1r
Hfe--Tfrc
Hbegf--Egfr
Glutamate-byGLS-and-SLC1A3--Grm5
Glutamate-byGLS-and-SLC1A3--Grm3
Glutamate-byGLS-and-SLC1A3--Gria2
Fn1--integrin-aVb1-complex
Fn1--integrin-a5b1-complex
Dkk2--Lrp6
Cxcl12--Cxcr4
Cxcl1--Dpp4
Col8a1--integrin-a1b1-complex
Col7a1--integrin-a1b1-complex
Col6a2--integrin-a1b1-complex
Col5a3--integrin-a1b1-complex
Col5a2--integrin-a1b1-complex
Col4a5--integrin-a1b1-complex
Col4a5--Adgrg6
Col4a2--integrin-a1b1-complex
Col4a2--Adgrg6
Col4a1--integrin-a1b1-complex
Col4a1--Adgrg6
Col3a1--integrin-a1b1-complex
Col3a1--Adgrg1
Col27a1--integrin-a1b1-complex
Col26a1--integrin-a1b1-complex
Col1a2--integrin-a1b1-complex
Col18a1--integrin-a1b1-complex
Cholesterol-byLIPA--Rora
Cd248--Il6ra
Bmp5--BMR1B-AVR2A
Bmp5--BMR1A-AVR2B
Bmp5--BMR1A-ACR2A
Bmp5--BMPR1B-BMPR2
Bmp5--BMPR1A-BMPR2
Bmp5--ACVR1-BMPR2
Bmp5--ACVR-1A2B-receptor
Bmp5--ACVR-1A2A-receptor
Bmp4--BMR1B-AVR2A
Bmp4--BMR1A-AVR2B
Bmp4--BMR1A-ACR2A
Bmp4--BMPR1B-BMPR2
Bmp4--BMPR1A-BMPR2
App--Sorl1
Angpt1--Tek
Agrn--Ptprs
Agrn--Ncam1
2arachidonoylglycerol-byDAGLA--Cnr1
Pericyte--Activated Microglia
Pericyte--Arterial
Pericyte--Astrocyte
Pericyte--Capillary
Pericyte--Microglia
Pericyte--Proliferating Microglia
Pericyte--Reactive Astrocyte
Pericyte--Venous
3.5
3.0
2.5
2.0
1.5
1.0
0.5
0.0
Mean L+R expression
P-value
0.001
0.01
0.1
1
b
Nodal--ACVR2B
Nodal--ACVR2A
Nodal--ACVR1B
Pericyte--Activated Microglia
Pericyte--Arterial
Pericyte--Astrocyte
Pericyte--Capillary
Pericyte--Microglia
Pericyte--Proliferating Microglia
Pericyte--Reactive Astrocyte
Pericyte--Venous
0.4
0.2
0.0
Mean L+R expression
P-value
0.001
0.01
0.1
1

Supplementary Figure 1. Characterization of pericyte-specific pathways in the postnatal brain.

(a, b) Dot plots showing predicted interactions between brain pericytes and other cell types based on CellPhoneDB analysis of P12 brain scRNA-seq data. Interactions mediated by brain pericyte-derived secreted factors **(a)** and Nodal signaling **(b)** are shown.

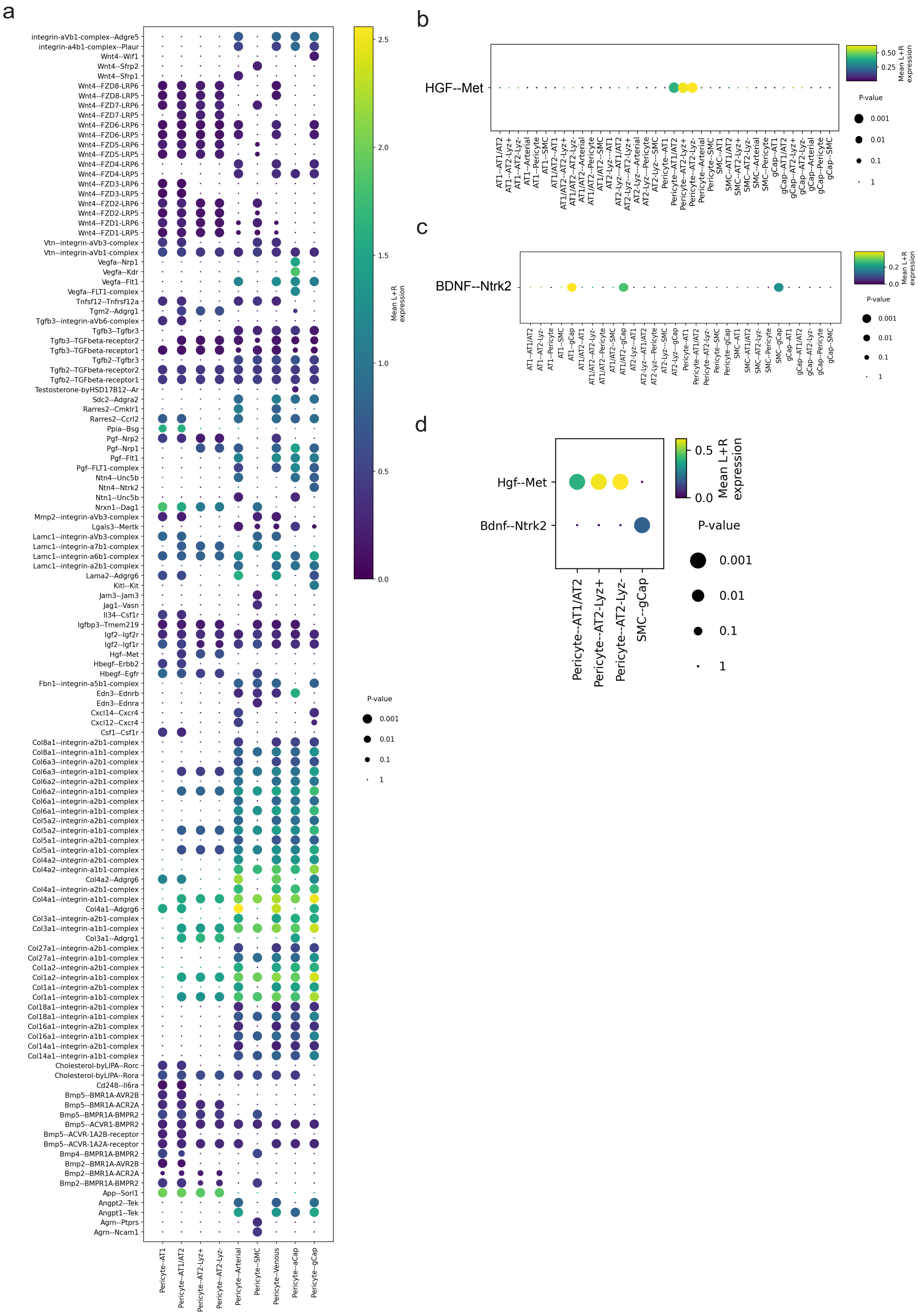
a
integrin-aVb1-complex--Adgre5
integrin-a4b1-complex--Plaur
Wnt4--Wif1
Wnt4--Sfrp2
Wnt4--Sfrp1
Wnt4--FZD8-LRP6
Wnt4--FZD8-LRP5
Wnt4--FZD7-LRP6
Wnt4--FZD7-LRP5
Wnt4--FZD6-LRP6
Wnt4--FZD6-LRP5
Wnt4--FZD5-LRP6
Wnt4--FZD5-LRP5
Wnt4--FZD4-LRP6
Wnt4--FZD4-LRP5
Wnt4--FZD3-LRP6
Wnt4--FZD3-LRP5
Wnt4--FZD2-LRP6
Wnt4--FZD2-LRP5
Wnt4--FZD1-LRP6
Wnt4--FZD1-LRP5
Vtn--integrin-aVb3-complex
Vtn--integrin-aVb1-complex
Vegfa--Nrp1
Vegfa--Kdr
Vegfa--Flt1
Vegfa--FLT1-complex
Tnfsf12--Tnfrsf12a
Tgm2--Adgrg1
Tgfb3--integrin-aVb6-complex
Tgfb3--Tgfbr3
Tgfb3--TGFbeta-receptor2
Tgfb3--TGFbeta-receptor1
Tgfb2--Tgfbr3
Tgfb2--TGFbeta-receptor2
Tgfb2--TGFbeta-receptor1
Testosterone-byHSD17B12--Ar
Sdc2--Adgra2
Rarres2--Cmklr1
Rarres2--Ccrl2
Ppia--Bsg
Pgf--Nrp2
Pgf--Nrp1
Pgf--Flt1
Pgf--FLT1-complex
Ntn4--Unc5b
Ntn4--Ntrk2
Ntn1--Unc5b
Nrxn1--Dag1
Mmp2--integrin-aVb3-complex
Lgals3--Mertk
Lamc1--integrin-aVb3-complex
Lamc1--integrin-a7b1-complex
Lamc1--integrin-a6b1-complex
Lamc1--integrin-a2b1-complex
Lama2--Adgrg6
Kitl--Kit
Jam3--Jam3
Jag1--Vasn
Il34--Csf1r
Igfbp3--Tmem219
Igf2--Igf2r
Igf2--Igf1r
Hgf--Met
Hbegf--Erbb2
Hbegf--Egfr
Fbn1--integrin-a5b1-complex
Edn3--Ednrb
Edn3--Ednra
Cxcl14--Cxcr4
Cxcl12--Cxcr4
Csf1--Csf1r
Col8a1--integrin-a2b1-complex
Col8a1--integrin-a1b1-complex
Col6a3--integrin-a2b1-complex
Col6a3--integrin-a1b1-complex
Col6a2--integrin-a2b1-complex
Col6a2--integrin-a1b1-complex
Col6a1--integrin-a2b1-complex
Col6a1--integrin-a1b1-complex
Col5a2--integrin-a2b1-complex
Col5a2--integrin-a1b1-complex
Col5a1--integrin-a2b1-complex
Col5a1--integrin-a1b1-complex
Col4a2--integrin-a2b1-complex
Col4a2--integrin-a1b1-complex
Col4a2--Adgrg6
Col4a1--integrin-a2b1-complex
Col4a1--integrin-a1b1-complex
Col4a1--Adgrg6
Col3a1--integrin-a2b1-complex
Col3a1--integrin-a1b1-complex
Col3a1--Adgrg1
Col27a1--integrin-a2b1-complex
Col27a1--integrin-a1b1-complex
Col1a2--integrin-a2b1-complex
Col1a2--integrin-a1b1-complex
Col1a1--integrin-a2b1-complex
Col1a1--integrin-a1b1-complex
Col18a1--integrin-a2b1-complex
Col18a1--integrin-a1b1-complex
Col16a1--integrin-a2b1-complex
Col16a1--integrin-a1b1-complex
Col14a1--integrin-a2b1-complex
Col14a1--integrin-a1b1-complex
Cholesterol-byLIPA--Rorc
Cholesterol-byLIPA--Rora
Cd248--Il6ra
Bmp5--BMR1A-AVR2B
Bmp5--BMR1A-ACR2A
Bmp5--BMPR1A-BMPR2
Bmp5--ACVR1-BMPR2
Bmp5--ACVR-1A2B-receptor
Bmp5--ACVR-1A2A-receptor
Bmp4--BMPR1A-BMPR2
Bmp2--BMR1A-AVR2B
Bmp2--BMR1A-ACR2A
Bmp2--BMPR1A-BMPR2
App--Sorl1
Angpt2--Tek
Angpt1--Tek
Agrn--Ptprs
Agrn--Ncam1
Pericyte--AT1
Pericyte--AT1/AT2
Pericyte--AT2-Lyz+
Pericyte--AT2-Lyz-
Pericyte--Arterial
Pericyte--SMC
Pericyte--Venous
Pericyte--aCap
Pericyte--gCap
2.5
2.0
1.5
1.0
0.5
0.0
Mean L+R expression
P-value
0.001
0.01
0.1
1
b
HGF--Met
AT1--AT1/AT2
AT1--AT2-Lyz+
AT1--AT2-Lyz-
AT1--Arterial
AT1--Pericyte
AT1--SMC
AT1/AT2--AT1
AT1/AT2--AT2-Lyz+
AT1/AT2--AT2-Lyz-
AT1/AT2--Arterial
AT1/AT2--Pericyte
AT1/AT2--SMC
AT2-Lyz--AT1
AT2-Lyz--AT1/AT2
AT2-Lyz--AT2-Lyz+
AT2-Lyz--Arterial
AT2-Lyz--Pericyte
AT2-Lyz--SMC
Pericyte--AT1
Pericyte--AT1/AT2
Pericyte--AT2-Lyz+
Pericyte--AT2-Lyz-
Pericyte--Arterial
Pericyte--SMC
SMC--AT1
SMC--AT1/AT2
SMC--AT2-Lyz+
SMC--AT2-Lyz-
SMC--Arterial
SMC--Pericyte
gCap--AT1
gCap--AT1/AT2
gCap--AT2-Lyz+
gCap--AT2-Lyz-
gCap--Arterial
gCap--Pericyte
gCap--SMC
0.50
0.25
Mean L+R expression
P-value
0.001
0.01
0.1
1
c
BDNF--Ntrk2
AT1--AT1/AT2
AT1--AT2-Lyz-
AT1--Pericyte
AT1--SMC
AT1--gCap
AT1/AT2--AT2-Lyz-
AT1/AT2--Pericyte
AT1/AT2--SMC
AT1/AT2--gCap
AT2-Lyz--AT1
AT2-Lyz--AT1/AT2
AT2-Lyz--Pericyte
AT2-Lyz--SMC
AT2-Lyz--gCap
Pericyte--AT1
Pericyte--AT1/AT2
Pericyte--AT2-Lyz-
Pericyte--SMC
Pericyte--gCap
SMC--AT1
SMC--AT1/AT2
SMC--AT2-Lyz-
SMC--Pericyte
SMC--gCap
gCap--AT1
gCap--AT1/AT2
gCap--AT2-Lyz-
gCap--Pericyte
gCap--SMC
0.2
0.0
Mean L+R expression
P-value
0.001
0.01
0.1
1
d
Hgf--Met
Bdnf--Ntrk2
Pericyte--AT1/AT2
Pericyte--AT2-Lyz+
Pericyte--AT2-Lyz-
SMC--gCap
0.5
0.0
Mean L+R expression
P-value
0.001
0.01
0.1
1

Supplementary Figure 2. Characterization of pericyte-specific pathways in the postnatal lung.

(a, d) Dot plots showing predicted interactions between lung pericytes and other cell types based on CellPhoneDB analysis of P21 lung scRNA-seq data. Interactions mediated by lung pericyte-derived secreted factors **(a),** HGF **(b, d)** and BDNF signaling **(c, d)** are shown.

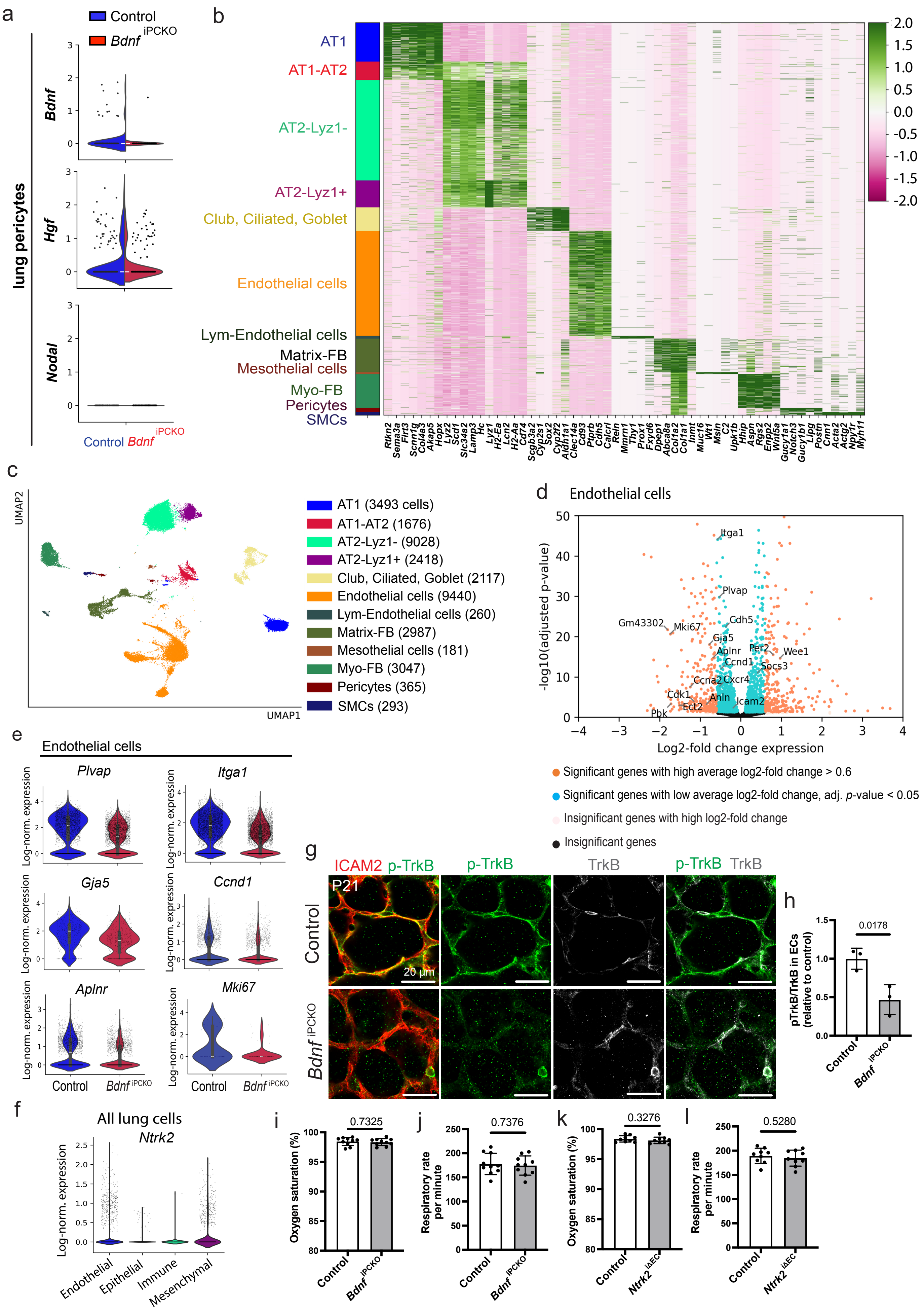
a
Control
Bdnf iPCKO
lung pericytes
Bdnf
Hgf
Nodal
Control Bdnf iPCKO
b
AT1
AT1-AT2
AT2-Lyz1-
AT2-Lyz1+
Club, Ciliated, Goblet
Endothelial cells
Lym-Endothelial cells
Matrix-FB
Mesothelial cells
Myo-FB
Pericytes
SMCs
c
UMAP2
UMAP1
AT1 (3493 cells)
AT1-AT2 (1676)
AT2-Lyz1- (9028)
AT2-Lyz1+ (2418)
Club, Ciliated, Goblet (2117)
Endothelial cells (9440)
Lym-Endothelial cells (260)
Matrix-FB (2987)
Mesothelial cells (181)
Myo-FB (3047)
Pericytes (365)
SMCs (293)
d
Endothelial cells
-log10(adjusted p-value)
Log2-fold change expression
Significant genes with high average log2-fold change > 0.6
Significant genes with low average log2-fold change, adj. p-value < 0.05
Insignificant genes with high log2-fold change
Insignificant genes
e
Endothelial cells
Plvap
Itga1
Gja5
Ccnd1
Aplnr
Mki67
Log-norm. expression
Control Bdnf iPCKO
f
All lung cells
Ntrk2
Log-norm. expression
Endothelial
Epithelial
Immune
Mesenchymal
g
ICAM2 p-TrkB
p-TrkB
TrkB
p-TrkB TrkB
P21
Control
Bdnf iPCKO
20 µm
h
pTrkB/TrkB in ECs (relative to control)
0.0178
Control
Bdnf iPCKO
i
Oxygen saturation (%)
0.7325
j
Respiratory rate per minute
0.7376
k
Oxygen saturation (%)
0.3276
Ntrk2 iΔEC
l
Respiratory rate per minute
0.5280

Supplementary Figure 3. scRNA-seq analysis of P21 *Bdnf*^{iPCKO} and control lungs.

(a) Violin plots showing efficient depletion of *Bdnf*, but not *Hgf* or *Nodal*, in lung pericytes from *Bdnf*^{iPCKO} relative to control animals. **(b)** Heatmap of top marker genes cell populations based on computational analysis of scRNA-seq data. **(c)** UMAP plot of annotated cell types from lung together with corresponding cell counts. Indicated are AT1 epithelial cells, AT1-AT2 transitional/intermediate cells, *Lyz1*+ AT2 and *Lyz1*- AT2 epithelial cells, Lymphatic endothelial cells, Matrix fibroblast, Myofibroblast, and smooth muscle cells.

(d) Volcano plot showing the differential expression analysis in P21 *Bdnf*^{iPCKO} and littermate control pulmonary ECs. Average log2-fold change > 0.6, adjusted p-value < 0.05). Pseudobulk DE analysis uses two-sided Wald test + independent filtering as implemented by pyDESeq2.

(e) Violin plots showing expression of multiple endothelial markers (*Plvap*, *Gja5*, *Aplnr* and Itga1), and proliferation markers (*Ccnd1* and *Mki67*) in pulmonary ECs. Boxplots in centre of violins shows median (white bar), quartiles (box), and the farthest data point within 1.5 * inter-quartile range (whiskers).

(f) Violin plot of *Ntrk2* expression in endothelial, epithelial, immune, and mesenchymal cells (including fibroblasts) from lung.

(g) Confocal images of pulmonary vasculature stained with ICAM2 (red), p-TrkB (green), TrkB (grey) from *Bdnf*^{iPCKO} and littermate control animals.

(h) Graph shows ratio of p-TrkB/TrkB expression in endothelial cells. Data represents mean ± s.e.m. (n=3); P-values, unpaired two tailed student t-test.

(i-l) Quantitation of arterial oxygen saturation (**i, k**) and respiratory rate (**j, l**) in P21 *Bdnf*^{iPCKO} (**i-j**), and *Ntrk2*$^{i\Delta EC}$ (**k, l**) mutants compared to littermate control mice. Data represents mean± s.e.m. (n=10 in (**i, j**) and n=9 in (**k, l**); P-values, unpaired two tailed student t-test.

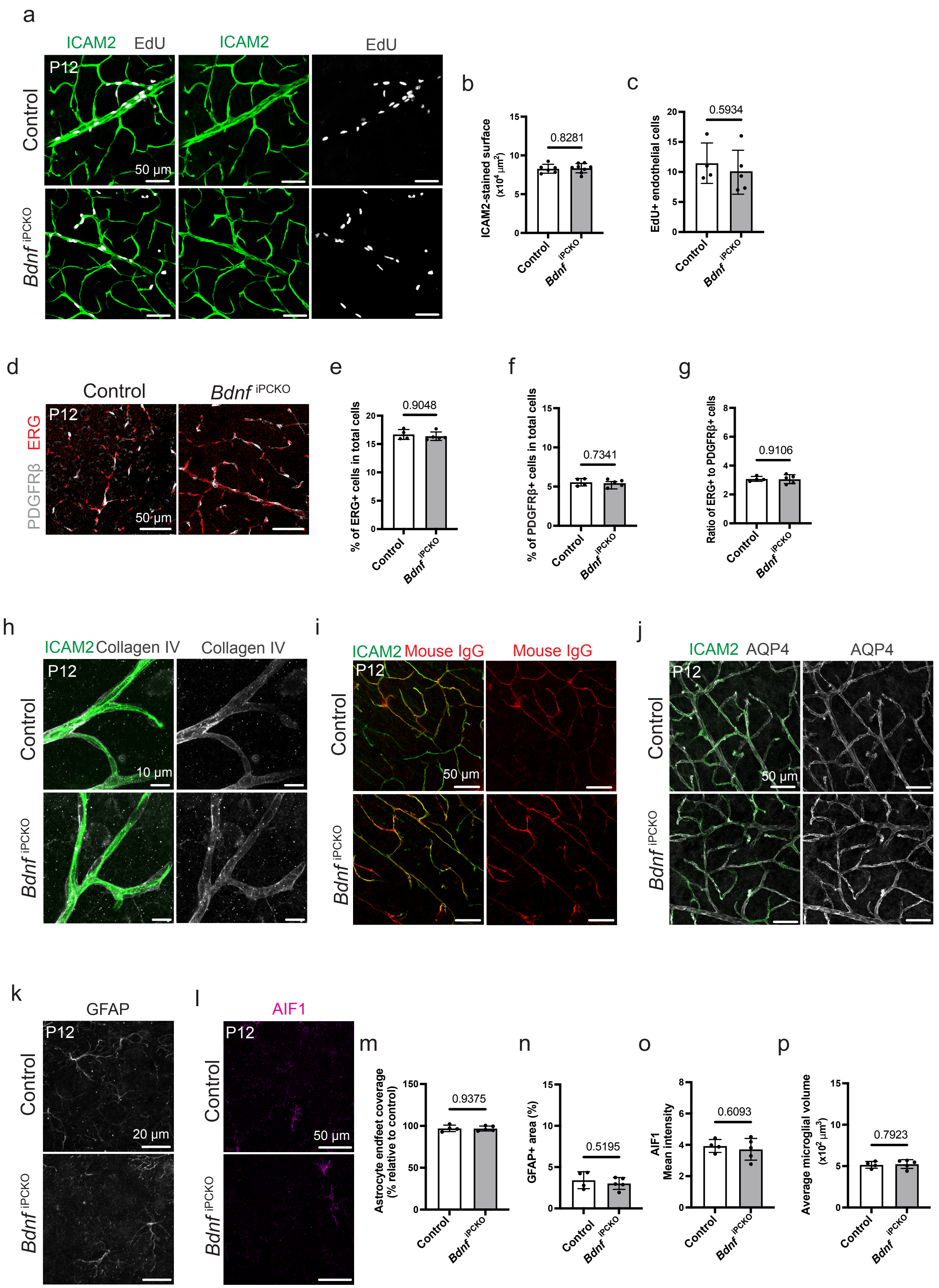

a
ICAM2 EdU
ICAM2
EdU
P12
Control
50 µm
Bdnf iPCKO
b
ICAM2-stained surface (x10^4 µm^2)
0.8281
Control
Bdnf iPCKO
c
EdU+ endothelial cells
0.5934
Control
Bdnf iPCKO
d
Control
Bdnf iPCKO
P12
ERG
PDGFRβ
50 µm
e
% of ERG+ cells in total cells
0.9048
Control
Bdnf iPCKO
f
% of PDGFRβ+ cells in total cells
0.7341
Control
Bdnf iPCKO
g
Ratio of ERG+ to PDGFRβ+ cells
0.9106
Control
Bdnf iPCKO
h
ICAM2 Collagen IV
Collagen IV
P12
Control
10 µm
Bdnf iPCKO
i
ICAM2 Mouse IgG
Mouse IgG
P12
Control
50 µm
Bdnf iPCKO
j
ICAM2 AQP4
AQP4
P12
Control
50 µm
Bdnf iPCKO
k
GFAP
P12
Control
20 µm
Bdnf iPCKO
l
AIF1
P12
Control
50 µm
Bdnf iPCKO
m
Astrocyte endfeet coverage (% relative to control)
0.9375
Control
Bdnf iPCKO
n
GFAP+ area (%)
0.5195
Control
Bdnf iPCKO
o
AIF1 Mean intensity
0.6093
Control
Bdnf iPCKO
p
Average microglial volume (x10^2 µm^3)
0.7923
Control
Bdnf iPCKO

Supplementary Figure 4. Analysis of the P12 *Bdnf* ^{iPCKO} brain vasculature.

(a) Confocal images of brain vasculature stained with ICAM2 (green) and EdU (white).

(b, c) Graphs showing the ICAM2+ vascular density **(b)** and number of EdU+ ECs **(c)** in *Bdnf* ^{iPCKO} and littermate control brain cortex. Data represents mean ± s.e.m. (n=6 controls and 8 mutants in **b**, n=4 controls and 5 mutants in **c**); P-values, unpaired two tailed student t-test.

(d) Maximum intensity projections of P12 *Bdnf* ^{iPCKO} and littermate control brain sections. Images show PDGFRβ+ pericytes (grey) and ERG+ (red) ECs.

(e-g) Graphs showing the ratio of ERG+ EC nuclei to total cells **(e)**, ratio of PDGFRβ+ cells to total cells **(f)**, ratio of ERG+ cells to PDGFRβ+ cells **(g)** in P12 *Bdnf* ^{iPCKO} and littermate control brain cortex. Data represents mean ± s.e.m. (n= 4 controls and 5 mutants in **e-g**); P-values, unpaired two tailed Mann-Whitney test in **e** and unpaired two tailed student t-test in **f, g**.

(h) *Bdnf* ^{iPCKO} and control brain vasculature stained for ICAM2 (green) and Collagen IV (grey), as indicated.

(i) Confocal images of *Bdnf* ^{iPCKO} and littermate controls cortex stained for ICAM2 (green) and mouse IgG (red). **(j)** Astrocyte endfeet covering ICAM2+ endothelial cells (green) are visualized with AQP4 (grey). **(k, l)** Confocal images of brain cortex showing no change in the activation of GFAP+ astrocytes (grey) **(k)** or AIF+ microglia (magenta) **(l)**. **(m-p)** Quantitation of astrocyte endfeet coverage in P12 cortical blood vessels **(m)**, GFAP+ area **(n)**, expression of the microglia-specific marker AIF1 **(o)**, and average microglial volume **(p)** in the *Bdnf* ^{iPCKO} and littermate control brain cortex. P-values, unpaired two tailed t-test, (n= 4 controls and 5 mutants in **m-p**).

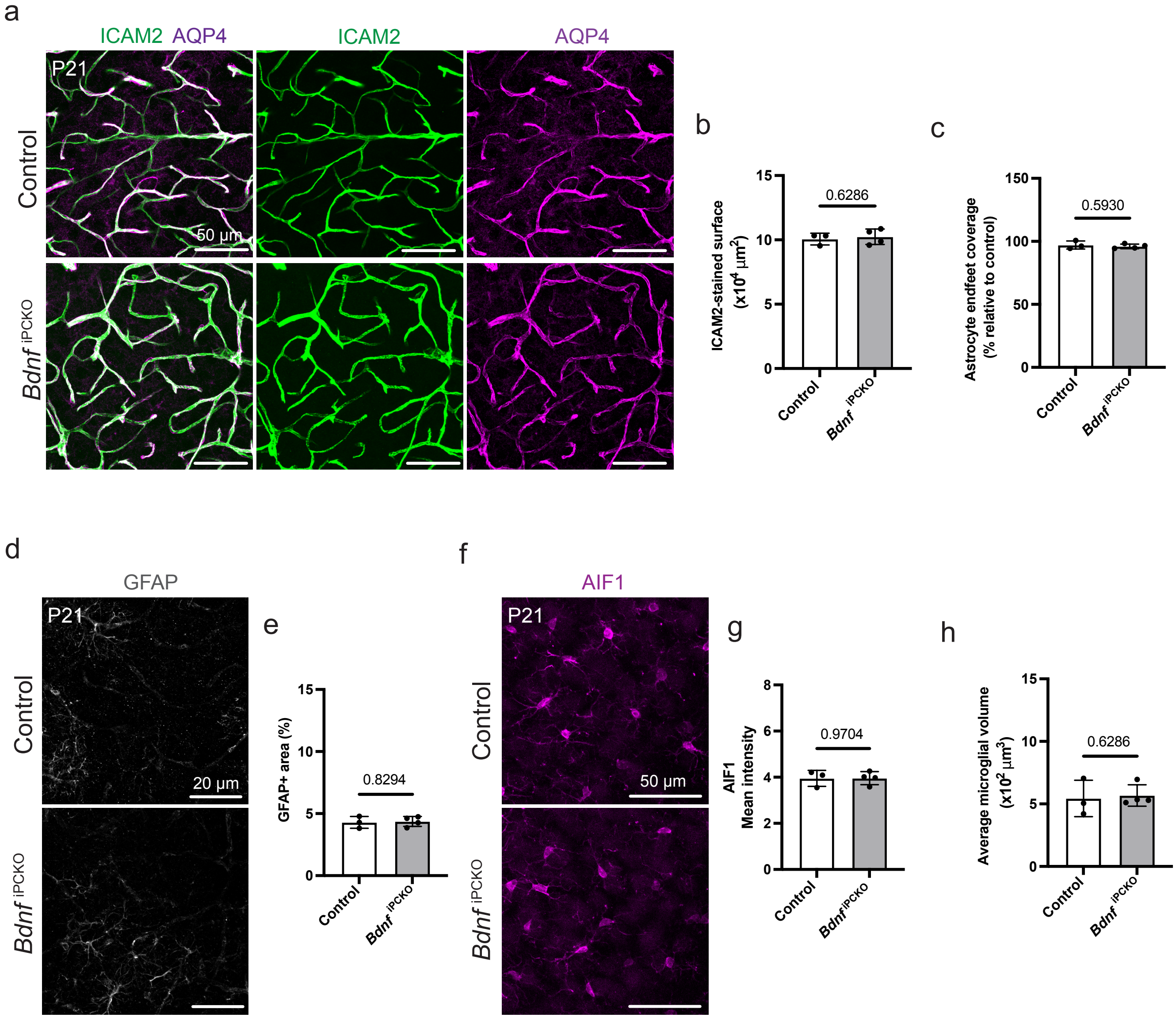
a
ICAM2 AQP4
ICAM2
AQP4
P21
Control
Bdnf iPCKO
50 µm
b
ICAM2-stained surface (x10^4 µm^2)
0.6286
Control
Bdnf iPCKO
c
Astrocyte endfeet coverage (% relative to control)
0.5930
Control
Bdnf iPCKO
d
GFAP
P21
Control
Bdnf iPCKO
20 µm
e
GFAP+ area (%)
0.8294
Control
Bdnf iPCKO
f
AIF1
P21
Control
Bdnf iPCKO
50 µm
g
AIF1 Mean intensity
0.9704
Control
Bdnf iPCKO
h
Average microglial volume (x10^2 µm^3)
0.6286
Control
Bdnf iPCKO

Supplementary Figure 5. Analysis of the P21 *Bdnf*^{iPCKO} brain vasculature.

(a) Confocal images of brain vasculature stained with ICAM2 (green) and AQP4 (magenta).

(b, c) Graphs showing the ICAM2+ vascular density **(b)** and astrocyte endfeet coverage in P21 cortical blood vessels **(c).** Data represents mean ± s.e.m. (n= 3 controls and 4 mutants in **b**, **c**); P-values, unpaired two tailed Mann-Whitney test in **b** and unpaired two tailed Student's t-test in **c**.

(d, f) Confocal images of brain cortex showing no change in the activation of GFAP+ astrocytes (grey) **(d)** or AIF+ microglia (magenta) **(f)**.

(e, g) Quantitation of GFAP+ area **(e)**, AIF1 expression **(g)**, and average microglial volume **(h)** in the *Bdnf*^{iPCKO} and littermate control brain cortex. Data represents mean ± s.e.m. (n= 3 controls and 4 mutants in **e**, **g** and **h**); P-values, unpaired two tailed Student's t-test in **e**, **g** and unpaired two tailed Mann-Whitney test in **h**.

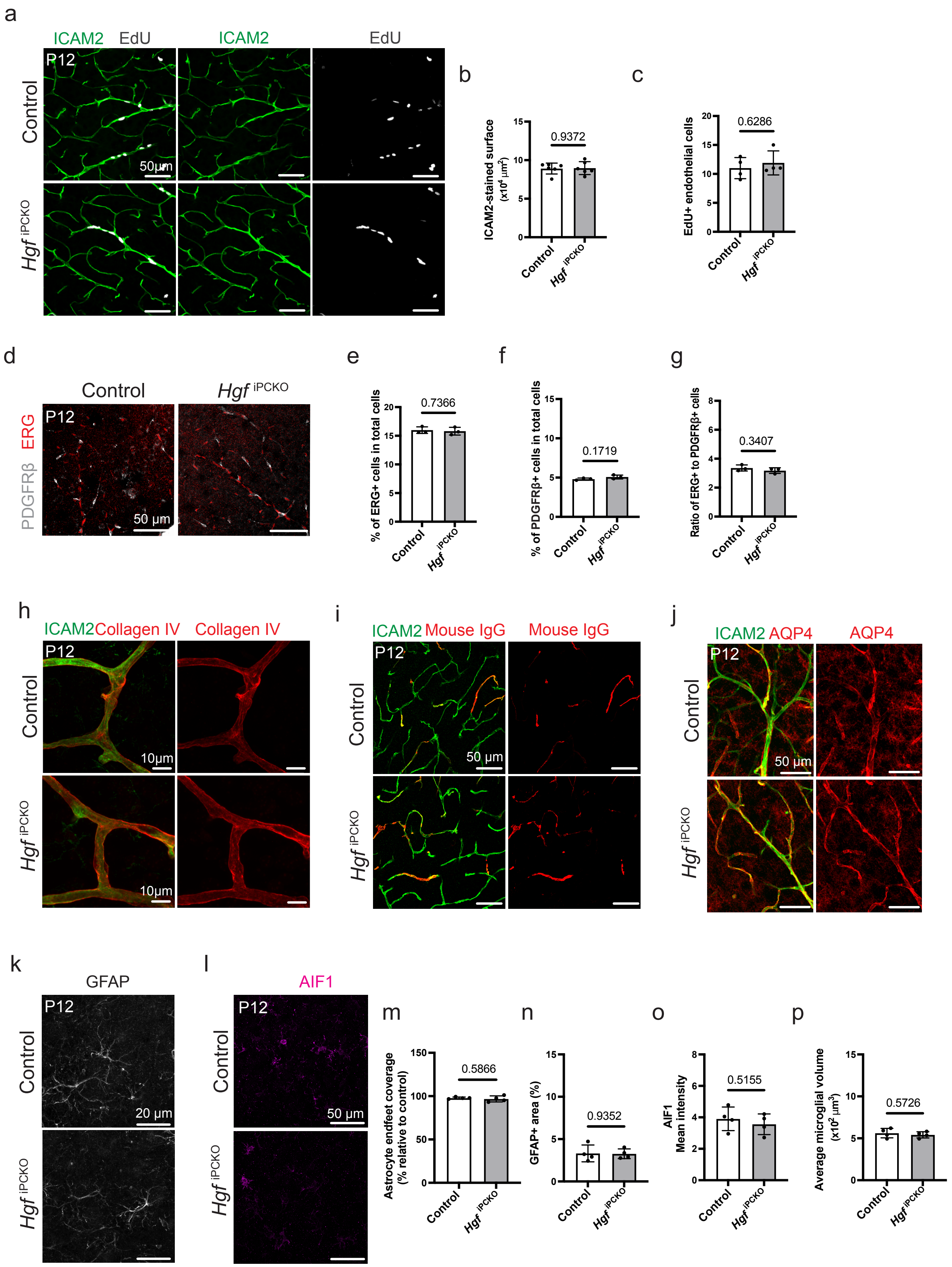

a
ICAM2 EdU
ICAM2
EdU
P12
Control
Hgf iPCKO
50µm
b
ICAM2-stained surface (x10⁴ µm²)
0.9372
Control
Hgf iPCKO
c
EdU+ endothelial cells
0.6286
Control
Hgf iPCKO
d
Control
Hgf iPCKO
P12
ERG
PDGFRβ
50 µm
e
% of ERG+ cells in total cells
0.7366
Control
Hgf iPCKO
f
% of PDGFRβ+ cells in total cells
0.1719
Control
Hgf iPCKO
g
Ratio of ERG+ to PDGFRβ+ cells
0.3407
Control
Hgf iPCKO
h
ICAM2 Collagen IV
Collagen IV
P12
Control
Hgf iPCKO
10µm
i
ICAM2 Mouse IgG
Mouse IgG
P12
Control
Hgf iPCKO
50 µm
j
ICAM2 AQP4
AQP4
P12
Control
Hgf iPCKO
50 µm
k
GFAP
P12
Control
Hgf iPCKO
20 µm
l
AIF1
P12
Control
Hgf iPCKO
50 µm
m
Astrocyte endfeet coverage (% relative to control)
0.5866
Control
Hgf iPCKO
n
GFAP+ area (%)
0.9352
Control
Hgf iPCKO
o
AIF1 Mean intensity
0.5155
Control
Hgf iPCKO
p
Average microglial volume (x10² µm³)
0.5726
Control
Hgf iPCKO

Supplementary Figure 6. Analysis of the P12 *Hgf*^{iPCKO} brain vasculature.

(a) Confocal images of brain vasculature stained with ICAM2 (green) and EdU (white).

(b, c) Graphs showing the ICAM2+ vascular density **(b)** and number of EdU+ ECs **(c)** in *Hgf* ^{iPCKO} and littermate control brain cortex. Data represents mean ± s.e.m. (n=6 in **b**, n=4 in **c**); P-values, unpaired two tailed Mann-Whitney test.

(d) Maximum intensity projections of P12 *Hgf*^{iPCKO} and littermate control brain sections. Images show PDGFRβ+ pericytes (grey) and ERG+ (red) ECs. **(e-g)** Graphs showing the ratio of ERG+ EC nuclei to total cells **(e)**, ratio of PDGFRβ+ cells to total cells **(f)**, ratio of ERG+ cells to PDGFRβ+ cells **(g)** in P12 *Hgf*^{iPCKO} and littermate control brain cortex. Data represents mean ± s.e.m. (n=3 in **e-g**); P-values, unpaired two tailed student t-test.

(h) *Hgf*^{iPCKO} and control brain vasculature stained for ICAM2 (green) and Collagen IV (grey), as indicated.

(i) Confocal images of *Hgf*^{iPCKO} and littermate controls cortex stained for ICAM2 (green) and mouse IgG (red).

(j) Astrocyte endfeet covering ICAM2+ endothelial cells (green) are visualized with AQP4 (grey).

(k, l) Confocal images of brain cortex showing no change in the activation of GFAP+ astrocytes (grey) **(k)** or AIF1+ microglia (magenta) **(l)**.

(m-p) Quantitation of astrocyte endfeet coverage in P12 cortical blood vessels **(m)**, GFAP+ area **(n)**, expression of AIF1 **(o)**, and average microglial volume **(p)** in the *Hgf*^{iPCKO} and littermate control brain cortex. Data represents mean ± s.e.m. P-values, unpaired two tailed Student's t-test, (n= 4 in **m-p**).

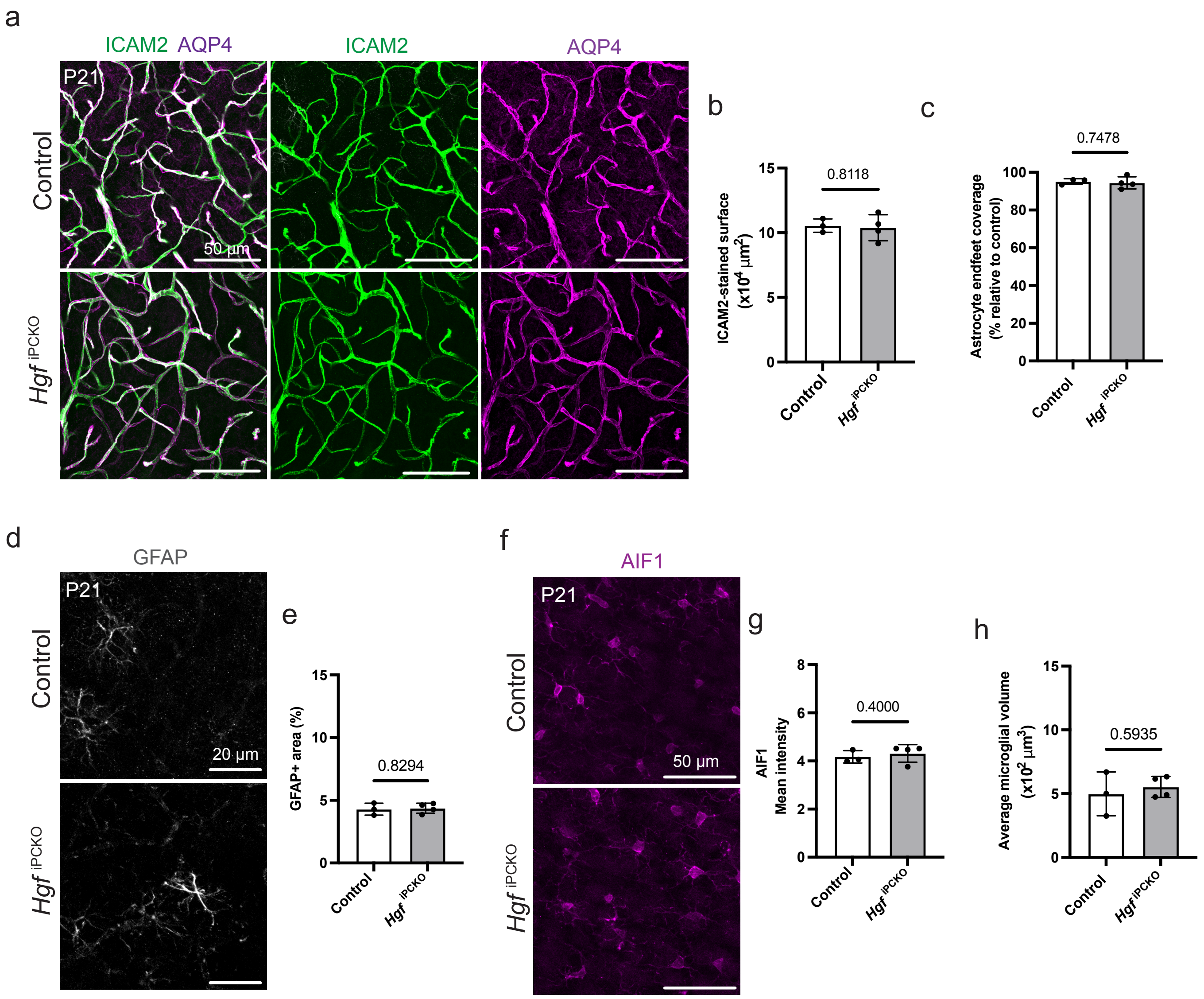

a
ICAM2 AQP4
ICAM2
AQP4
P21
Control
Hgf iPCKO
50 µm
b
ICAM2-stained surface (x10^4 µm^2)
0.8118
c
Astrocyte endfeet coverage (% relative to control)
0.7478
Control
Hgf iPCKO
d
GFAP
P21
20 µm
e
GFAP+ area (%)
0.8294
f
AIF1
P21
50 µm
g
AIF1 Mean intensity
0.4000
h
Average microglial volume (x10^2 µm^3)
0.5935

Supplementary Figure 7. Analysis of the P21 *Hgf*^{iPCKO} brain vasculature.

(a) Confocal images of brain vasculature stained with ICAM2 (green) and AQP4 (magenta).

(b, c) Graphs showing the ICAM2-stained vascular density **(b)** and astrocyte endfeet coverage in P21 cortical blood vessels **(c).**

(d, f) Confocal images of brain cortex showing no change in the activation of GFAP+ astrocytes (grey) **(d)** or AIF+ microglia (magenta) **(f)**.

(e-h) Quantitation of GFAP+ area **(e)**, AIF1 expression **(g)**, and average microglial volume **(h)** in the *Hgf*^{iPCKO} and littermate control brain cortex. Data represents mean ± s.e.m. (n= 3 controls and 4 mutants in **b**, **c**, **e**, **g** and **h**); P-values, unpaired two tailed Student's t-test in **b**, **c**, **e, h** and unpaired two tailed Mann-Whitney test in **g**.

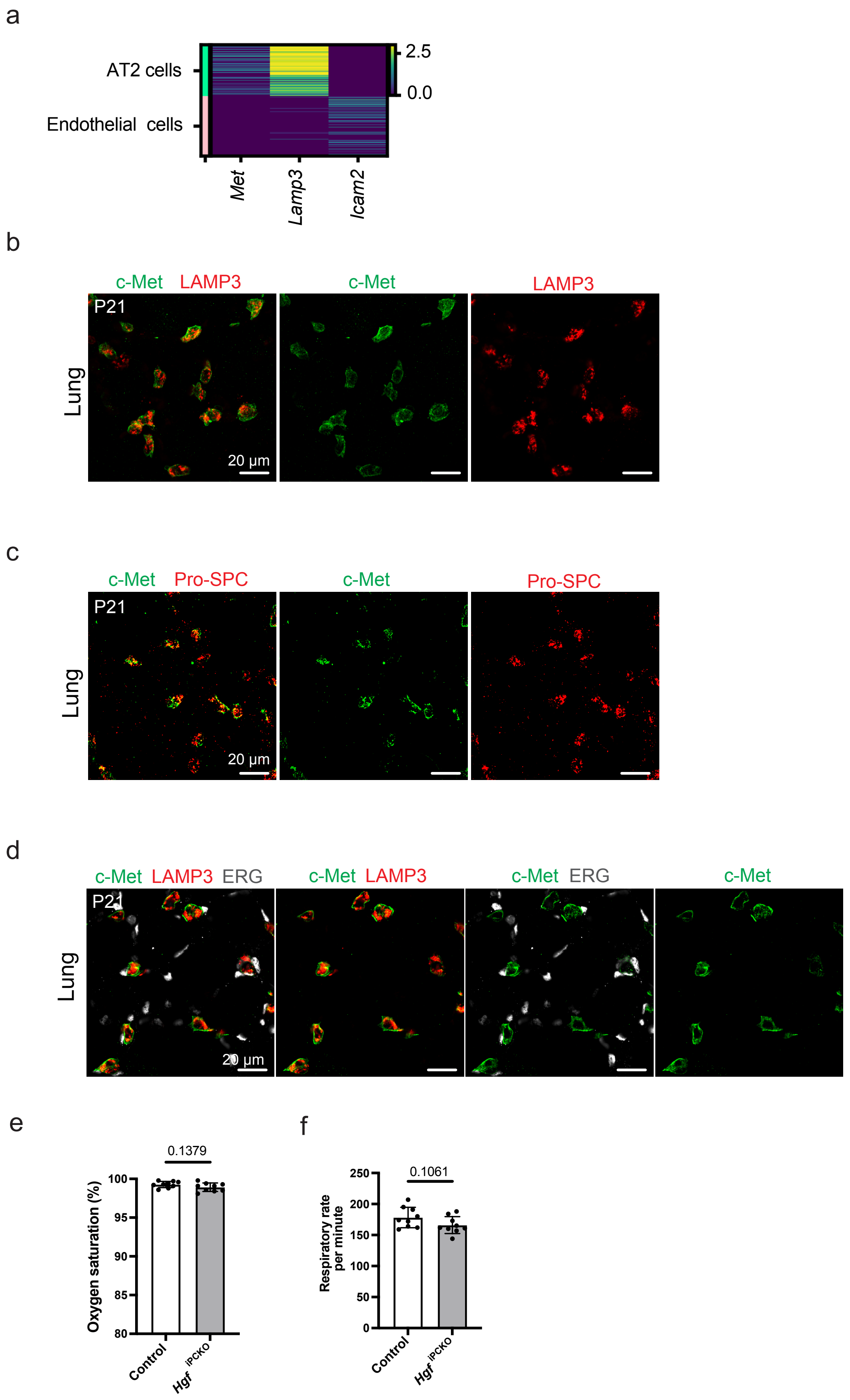

a
AT2 cells
Endothelial cells
Met
Lamp3
Icam2
2.5
0.0
b
c-Met LAMP3
c-Met
LAMP3
P21
Lung
20 μm
c
c-Met Pro-SPC
c-Met
Pro-SPC
P21
Lung
20 μm
d
c-Met LAMP3 ERG
c-Met LAMP3
c-Met ERG
c-Met
P21
Lung
20 μm
e
0.1379
Oxygen saturation (%)
100
95
90
85
80
Control
Hgf iPCKO
f
0.1061
Respiratory rate per minute
250
200
150
100
50
0
Control
Hgf iPCKO

Supplementary Figure 8. Expression of c-Met in AT2 epithelial cells but not ECs.

(a) Heatmap of *Met*, *Lamp3* and *Icam2* expression within AT2 and endothelial cells based on scRNA-seq results shows that *Met* is expressed by AT2 cells but not in endothelial cells.

(b-c) Confocal images of lungs showing that c-Met (green in **b** and **c**) is co-stained with AT2 markers including LAMP3+ **(**red in **b)** and Pro-SPC **(**red in **c)**.

(d) Confocal images of lung showing that c-Met (green) is co-stained with AT2 markers LAMP3+ AT2 cells (red) but not with ERG+ pulmonary endothelial cells (grey).

(e, f) Quantitation of arterial oxygen saturation (**e**) and respiratory rate (**f**) in P21 *Hgf*^{iPCKO} mutants compared to littermate controls. Data represents mean± s.e.m. (n=9 in **e** and **f**); P-values, unpaired two tailed student t-test.

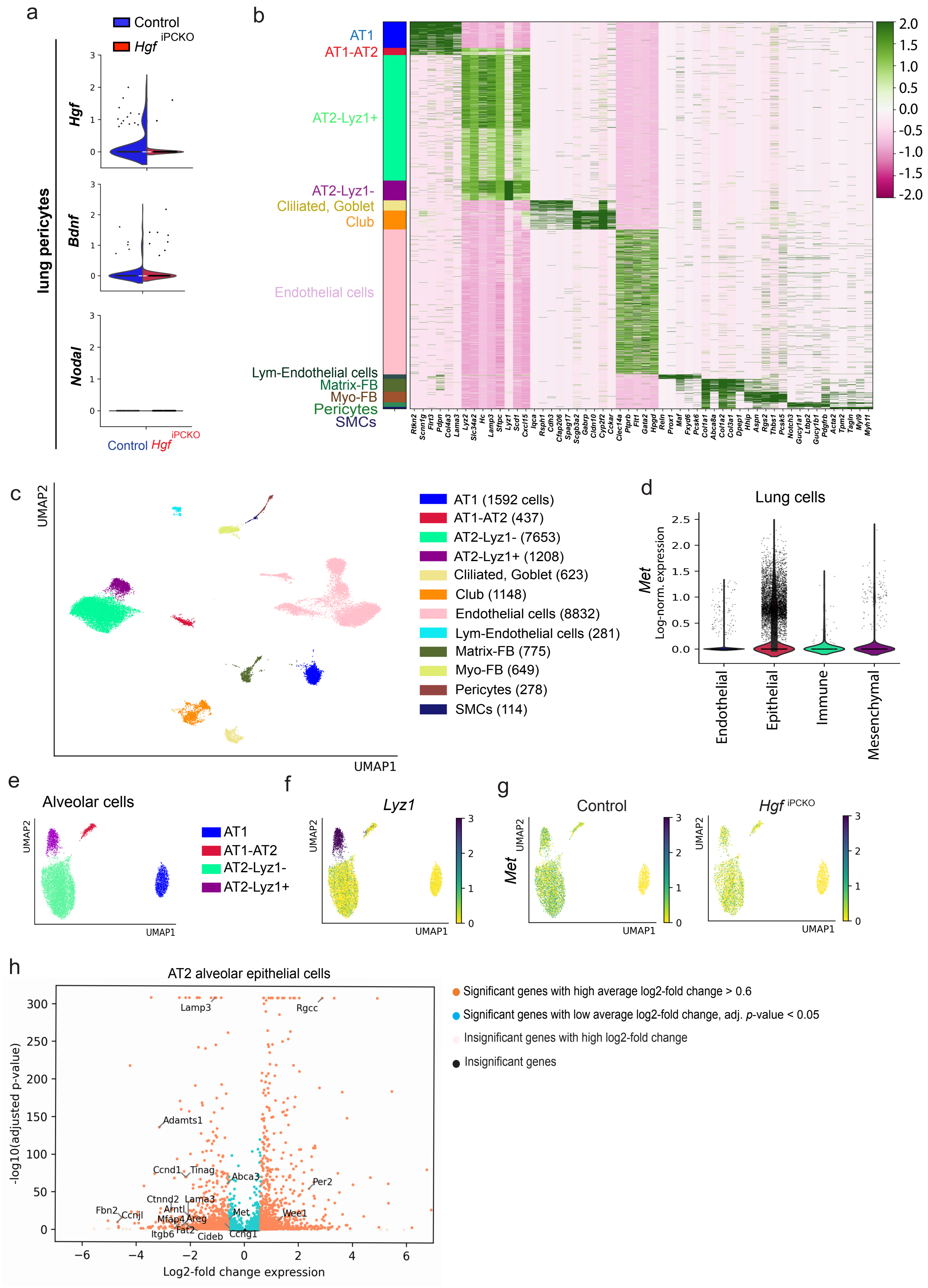

a
Control
Hgf iPCKO
lung pericytes
Hgf
Bdnf
Nodal
Control Hgf iPCKO
b
AT1
AT1-AT2
AT2-Lyz1+
AT2-Lyz1-
Cliliated, Goblet
Club
Endothelial cells
Lym-Endothelial cells
Matrix-FB
Myo-FB
Pericytes
SMCs
Rtkn2 Scnn1g Flrt3 Pdpn Col4a3 Lama3 Lyz2 Slc34a2 Hc Lamp3 Sftpc Lyz1 Scd1 Cxcl15 Iqca Rsph1 Cdh3 Cfap206 Spag17 Scgb3a2 Gabrp Cldn10 Cyp2f2 Cckar Clec14a Ptprb Flt1 Gata2 Hpgd Reln Prox1 Maf Fxyd6 Pcsk6 Col1a1 Abca8a Col1a2 Col3a1 Dpep1 Hhip Aspn Rgs2 Thbs1 Pcsk5 Notch3 Gucy1a1 Ltbp2 Gucy1b1 Pdgfrb Acta2 Tpm2 Tagln Myl9 Myh11
c
UMAP2
UMAP1
AT1 (1592 cells)
AT1-AT2 (437)
AT2-Lyz1- (7653)
AT2-Lyz1+ (1208)
Cliliated, Goblet (623)
Club (1148)
Endothelial cells (8832)
Lym-Endothelial cells (281)
Matrix-FB (775)
Myo-FB (649)
Pericytes (278)
SMCs (114)
d
Lung cells
Met Log-norm. expression
Endothelial
Epithelial
Immune
Mesenchymal
e
Alveolar cells
AT1
AT1-AT2
AT2-Lyz1-
AT2-Lyz1+
f
Lyz1
g
Control
Hgf iPCKO
Met
h
AT2 alveolar epithelial cells
-log10(adjusted p-value)
Log2-fold change expression
Lamp3
Rgcc
Adamts1
Ccnd1
Tinag
Abca3
Per2
Fbn2
Ccnjl
Ctnnd2
Lama3
Arntl
Mfap4
Areg
Met
Wee1
Itgb6
Fat2
Cideb
Ccng1
Significant genes with high average log2-fold change > 0.6
Significant genes with low average log2-fold change, adj. p-value < 0.05
Insignificant genes with high log2-fold change
Insignificant genes

Supplementary Figure 9. scRNA-seq analysis of P21 *Hgf* ^{iPCKO} and control lungs.

(a) Violin plots showing efficient depletion of *Hgf*, but not *Bdnf* or *Nodal*, in lung pericytes from *Hgf* ^{iPCKO} and control animals.

(b, c) Heatmap of top marker genes within lung cell types based on scRNA-seq results (**b**) and UMAP plot of annotated lung cell types with the corresponding cell counts (**c**). Indicated are AT1 epithelial cells, AT1-AT2 transitional/intermediate cells, *Lyz1*+ AT2 and *Lyz1*- AT2 epithelial cells, Lymphatic endothelial cells, Matrix fibroblast, Myofibroblast, and Smooth Muscle Cells (SMCs).

(d) Violin plot showing that *Met* expression dominates in epithelial cells relative to endothelial, immune and mesenchymal cells from P21 lung.

(e) UMAP plot of annotated alveolar cell types. Indicated are AT1 epithelial cells, AT1-AT2 transitional/intermediate cells, *Lyz1*+ AT2 and *Lyz1*- AT2 cells.

(f) UMAP plot showing *Lyz1* expression in a subcluster of AT2 epithelial cells.

(g) UMAP plots showing *Met* expression in AT1-AT2, *Lyz1*+ AT2 and *Lyz1*- AT2 cells from *Hgf* ^{iPCKO} and littermate control lungs.

(h) Volcano plot showing the differential expression analysis of endothelial-specific responses in *Hgf* ^{iPCKO} and littermate control lungs. Average log2-fold change > 0.6, adjusted p-value < 0.05). Pseudobulk DE analysis uses two-sided Wald test + independent filtering as implemented by pyDESeq2.

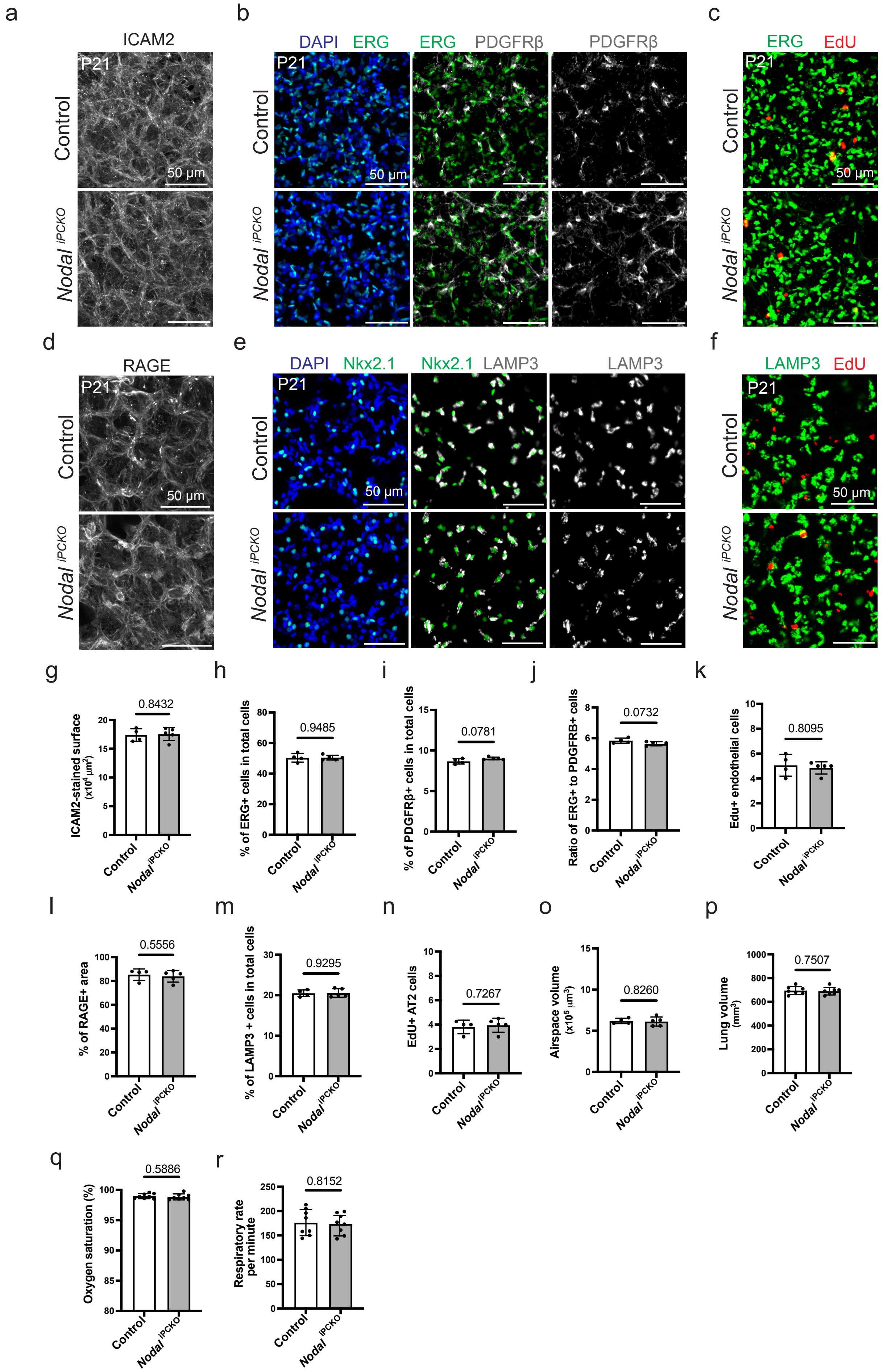

a
ICAM2
P21
Control
Nodal iPCKO
50 µm
b
DAPI ERG
ERG PDGFRβ
PDGFRβ
c
ERG EdU
d
RAGE
e
DAPI Nkx2.1
Nkx2.1 LAMP3
LAMP3
f
LAMP3 EdU
g
ICAM2-stained surface (x10^4 µm^2)
0.8432
h
% of ERG+ cells in total cells
0.9485
i
% of PDGFRβ+ cells in total cells
0.0781
j
Ratio of ERG+ to PDGFRB+ cells
0.0732
k
Edu+ endothelial cells
0.8095
l
% of RAGE+ area
0.5556
m
% of LAMP3 + cells in total cells
0.9295
n
EdU+ AT2 cells
0.7267
o
Airspace volume (x10^6 µm^3)
0.8260
p
Lung volume (mm^3)
0.7507
q
Oxygen saturation (%)
0.5886
r
Respiratory rate per minute
0.8152

Supplementary Figure 10. Normal lung development in *Nodal* ^{iPCKO} mutants.

(a, b) Confocal images of endothelium (ICAM2+, grey) **(a)**, EC nuclei (ERG+, green) and pericytes (PDGFRβ+, white) **(b)**, which show pulmonary vascular development is not affected in P21 *Nodal* ^{iPCKO} mutants.

(c) Confocal images of *Nodal* ^{iPCKO} and littermate control lungs showing EdU-labeled (red) ECs (ERG+, green).

(d, e) Maximum intensity projections of *Nodal* ^{iPCKO} and control lung sections showing AT1 cells (RAGE+, grey) **(d)**, nuclei of AT1 and AT2 cells (NKX2.1+, green), and type 2 alveolar epithelial cells (LAMP3+, white) **(e)**.

(f) Confocal image of proliferating cells (EdU+, red) and LAMP3-stained AT2 cells (green) in *Nodal* ^{iPCKO} and littermate control lungs.

(g-p) Graphs showing the ICAM2+ vascular density **(g)**, percentage of ERG+ cells in total cells **(h)**, percentage of PDGFRβ+ cells in total cells **(i)**, ratio of ERG+ to PDGFRβ+ cells **(j)**, the number of EdU+ ECs per area (283 x 283 x 22 μm) **(k)**, percentage of AT1 (RAGE+) area **(l)**, percentage of LAMP3+ AT2 cells in total cells **(m)**, the number of proliferating cells (EdU+) in AT2 cells **(n)**, airspace volume **(o)**, and lung volume measurement **(p)** in P21 *Nodal* ^{iPCKO} and littermate control lungs. Data represent mean ± s.e.m. (n=4 controls and 5 mutants in **g-o**, n=7 in **p**); P-values, unpaired two tailed student t-test in **g-j**, **m-p** and unpaired two tailed Mann-Whitney test in **k**, **l**.

(q, r) Quantitation of arterial oxygen saturation (**q**) and respiratory rate (**r**) in P21 *Nodal* ^{iPCKO} mutants compared to littermate controls. Data represents mean± s.e.m. (n=8 in **q** and **r**); P-values, unpaired two tailed student t-test.

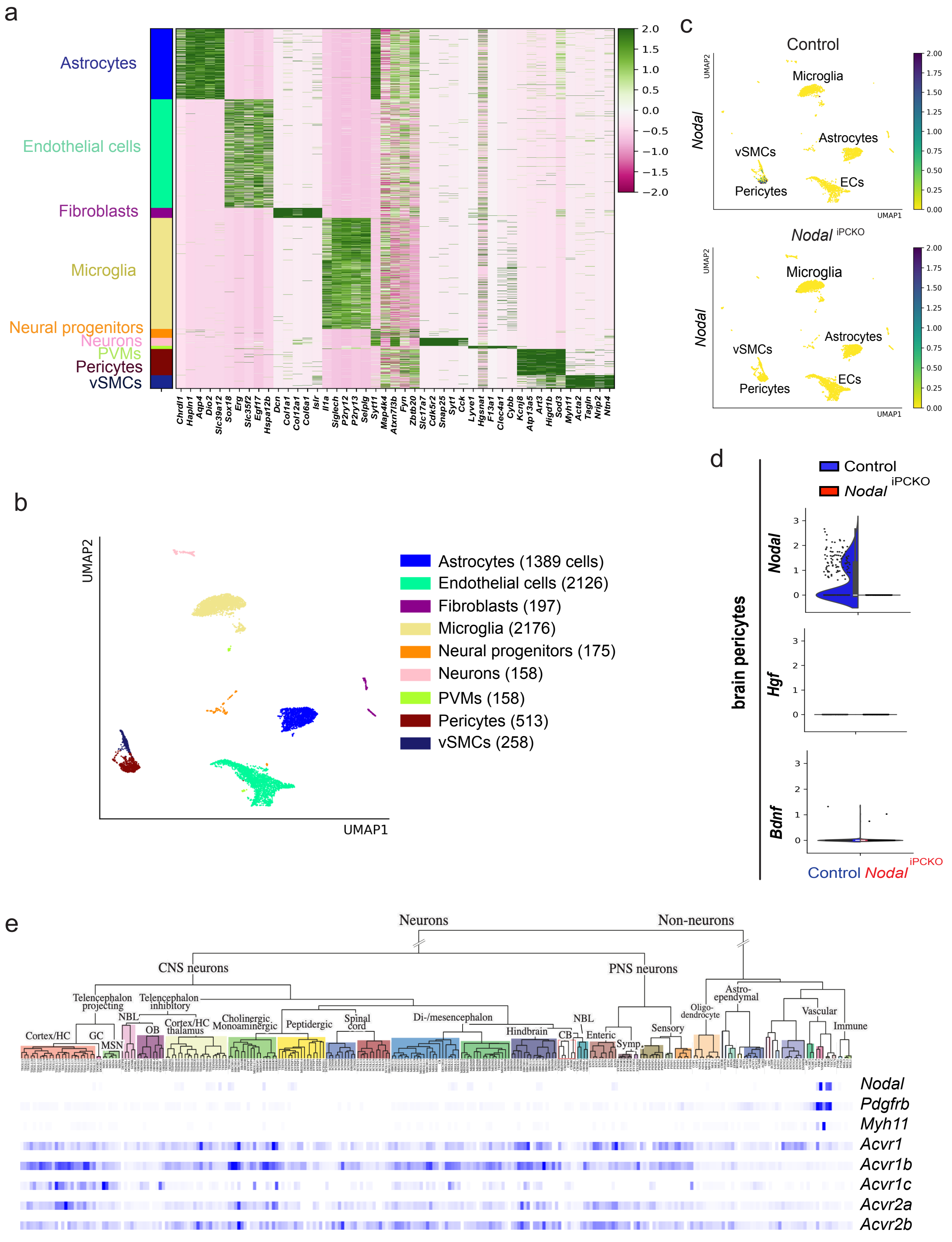

f

Name	Description	*Nodal*	*Pdgfrb*	*Myh11*	*Acvr1*	*Acvr1b*	*Acvr1c*	*Acvr2a*	*Acvr2b*
PER3	Pericytes	0.210	2.33	0.00598	0.0134	0.0100	0.00	0.00	0.00666
VSMCA	Vascular smooth muscle cells, arterial	0.00403	1.11	3.74	0.0245	0.0262	0.00334	0.0124	0.0129
PER1	Pericytes	0.124	2.19	0.0165	0.0378	0.00480	0.000456	0.0212	0.0269
PER2	Pericytes, possibly mixed with VENC	0.138	2.58	0.0180	0.0655	0.00828	0.00	0.0102	0.0363

Supplementary Figure 11. scRNA-seq analysis of *Nodal* expression in brain.

(a) Heatmap of top marker genes within brain cell types using based on scRNA-seq data.

(b) UMAP plot of annotated brain cell types. Each cell type is represented by distinct clusters and the corresponding cell counts are indicated. Indicated are perivascular macrophages, and vascular smooth muscle cells (vSMCs).

(c) UMAP plots show enriched expression of *Nodal* in control brain pericytes relative to other cell types and loss of expression in *Nodal* ^{iPCKO} pericytes.

(d) Violin plots showing efficient depletion of *Nodal*, but not *Hgf* or *Bdnf*, in brain pericytes from *Nodal* ^{iPCKO} and control animals.

(e) The expression of *Nodal*, *Pdgfrb* and transcripts for TGFβ family receptors in published scRNA-seq data of postnatal mouse brain. Dendrogram (obtained from mousebrain.org) displaying the taxonomy of all identified cell types from postnatal mouse brain and expression of the indicated genes. Note presence of TGFβ family receptor transcripts in many cell types.

(f) Circle plots of scRNA-seq data of adolescent mouse brain[40] showing the expression of *Nodal*, *Pdgfrb, Myh11* and TGFβ family receptor transcripts in mural cell populations.

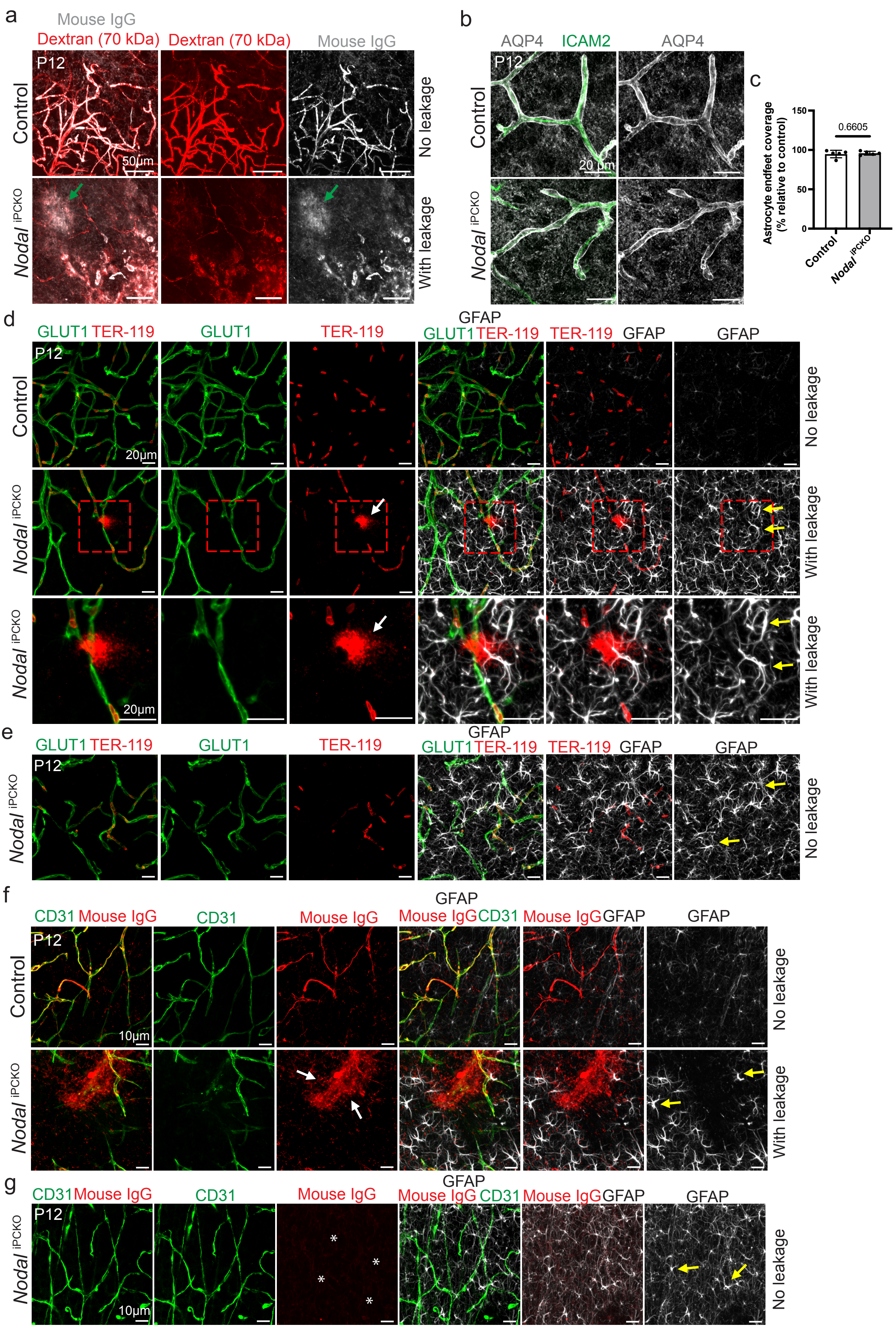
a
Mouse IgG
Dextran (70 kDa)
Dextran (70 kDa)
Mouse IgG
P12
Control
Nodal iPCKO
50µm
No leakage
With leakage
b
AQP4
ICAM2
AQP4
P12
Control
Nodal iPCKO
20 µm
c
Astrocyte endfeet coverage (% relative to control)
150
100
50
0
0.6605
Control
Nodal iPCKO
d
GLUT1 TER-119
GLUT1
TER-119
GFAP GLUT1 TER-119
TER-119 GFAP
GFAP
P12
Control
Nodal iPCKO
Nodal iPCKO
20µm
20µm
No leakage
With leakage
With leakage
e
GLUT1 TER-119
GLUT1
TER-119
GFAP GLUT1 TER-119
TER-119 GFAP
GFAP
P12
Nodal iPCKO
No leakage
f
CD31 Mouse IgG
CD31
Mouse IgG
GFAP Mouse IgG CD31
Mouse IgG GFAP
GFAP
P12
Control
Nodal iPCKO
10µm
No leakage
With leakage
g
CD31 Mouse IgG
CD31
Mouse IgG
GFAP Mouse IgG CD31
Mouse IgG GFAP
GFAP
P12
Nodal iPCKO
10µm
No leakage

Supplementary Figure 12. Characterization of *Nodal*^{iPCKO} brain capillaries.

(a) Confocal images of P12 brain cortex stained for mouse immunoglobulin G (IgG, white) from *Nodal*^{iPCKO} and control littermates injected with Texas Red-dextran (70 kDa) (red). Green arrows indicate local leakage.

(b) Maximum intensity projections showing comparable coverage of Aquaporin-4+ (AQP4+) astrocyte endfeet (grey) of ICAM2+ endothelium (green) in *Nodal*^{iPCKO} and control brain cortex.

(c) Quantitation of astrocyte endfeet coverage in P12 cortical blood vessels. Data represents mean± s.e.m. (n=5); P-values, unpaired two tailed student t-test.

(d, e) Confocal images of GLUT1+ (green) capillaries in brain cortex. Red blood cells (Ter119, red) are confined to vessels in control but leakage (arrows, white arrows) into the brain parenchyma and GFAP+ astrocytes (white, yellow arrowheads) can be seen in *Nodal*^{iPCKO} mutants **(d)**. Notably, reactive astrocytes (white, yellow arrows) are also observed in regions of the *Nodal*^{iPCKO} cortex without leakage **(e)**.

(f, g) Confocal images of CD31+ (green) brain capillaries with area of extravasated IgG (red, white arrows) in *Nodal*^{iPCKO} mutant surrounded by GFAP+ astrocytes (white, yellow arrows) in brain cortex (**f**). However, reactive astrocytes (yellow arrows) can also be observed in areas of *Nodal*^{iPCKO} cortex lacking extravasated IgG (asterisks) (**g**).

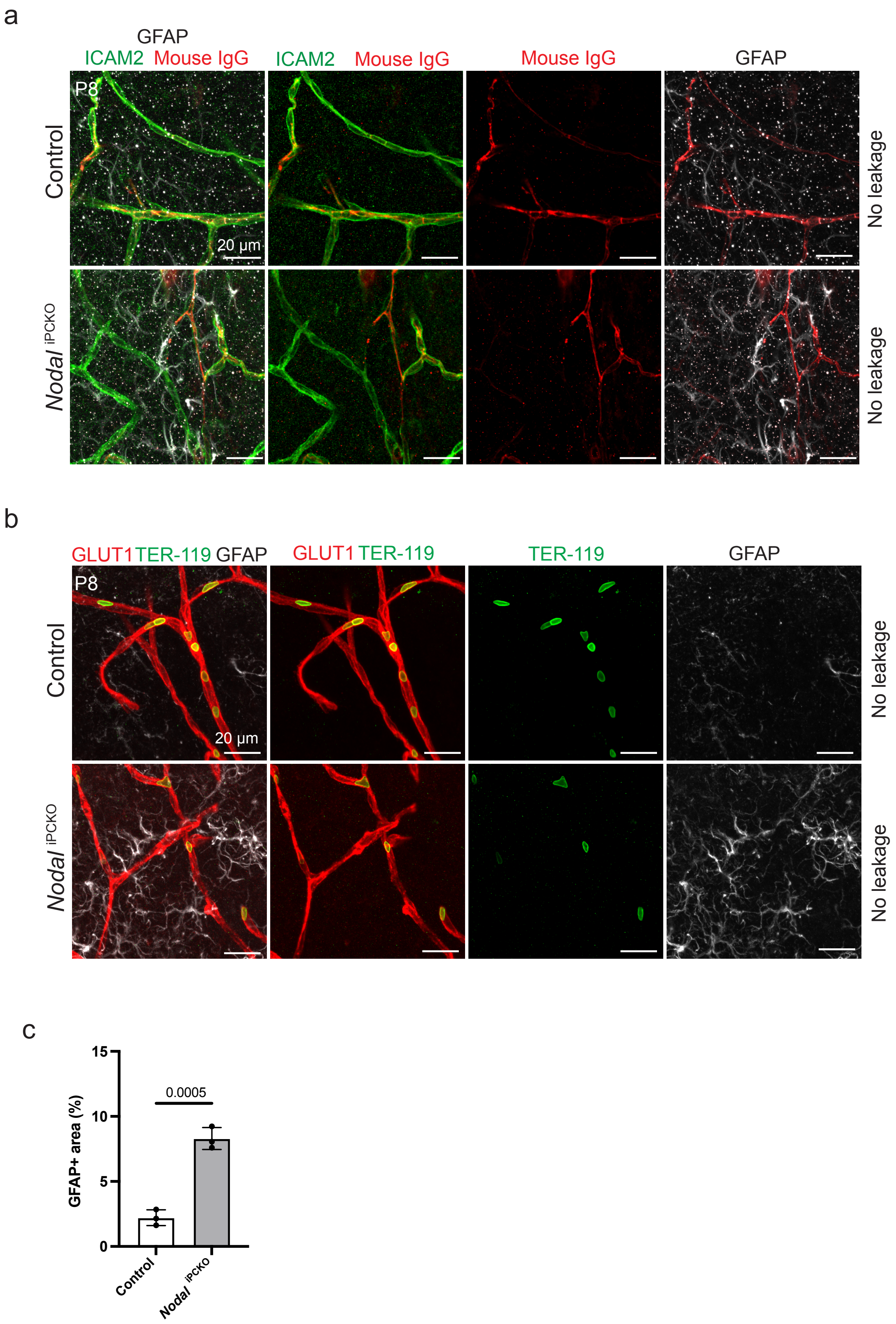

a
GFAP
ICAM2 Mouse IgG
ICAM2 Mouse IgG
Mouse IgG
GFAP
P8
Control
20 µm
No leakage
Nodal iPCKO
No leakage
b
GLUT1 TER-119 GFAP
GLUT1 TER-119
TER-119
GFAP
P8
Control
20 µm
No leakage
Nodal iPCKO
No leakage
c
GFAP+ area (%)
15
10
5
0
0.0005
Control
Nodal iPCKO

Supplementary Figure 13. Reactive astrogliosis emerges early in *Nodal* ^{iPCKO} cortex.

(a) Confocal images of brain cortex stained for ICAM2 (green) and mouse immunoglobulin G (IgG, red) showing lack of extravasated IgG in P8 *Nodal*^{iPCKO} and control littermates (3/3 brains), but GFAP+ astrocytes (white) can also be seen in *Nodal*^{iPCKO} mutants.

(b) Confocal images of GLUT1+ (red) capillaries in brain cortex. Red blood cells (Ter119, green) are confined to vessels in P8 *Nodal*^{iPCKO} and control littermates (3/3 brains), but GFAP+ astrocytes (white) can also be observed in *Nodal*^{iPCKO} mutants.

(c) Quantitation of GFAP+ area in the P8 *Nodal* ^{iPCKO} and littermate control brain cortex. Data represents mean ± s.e.m. (n=3); P-value, unpaired two tailed student t-test.

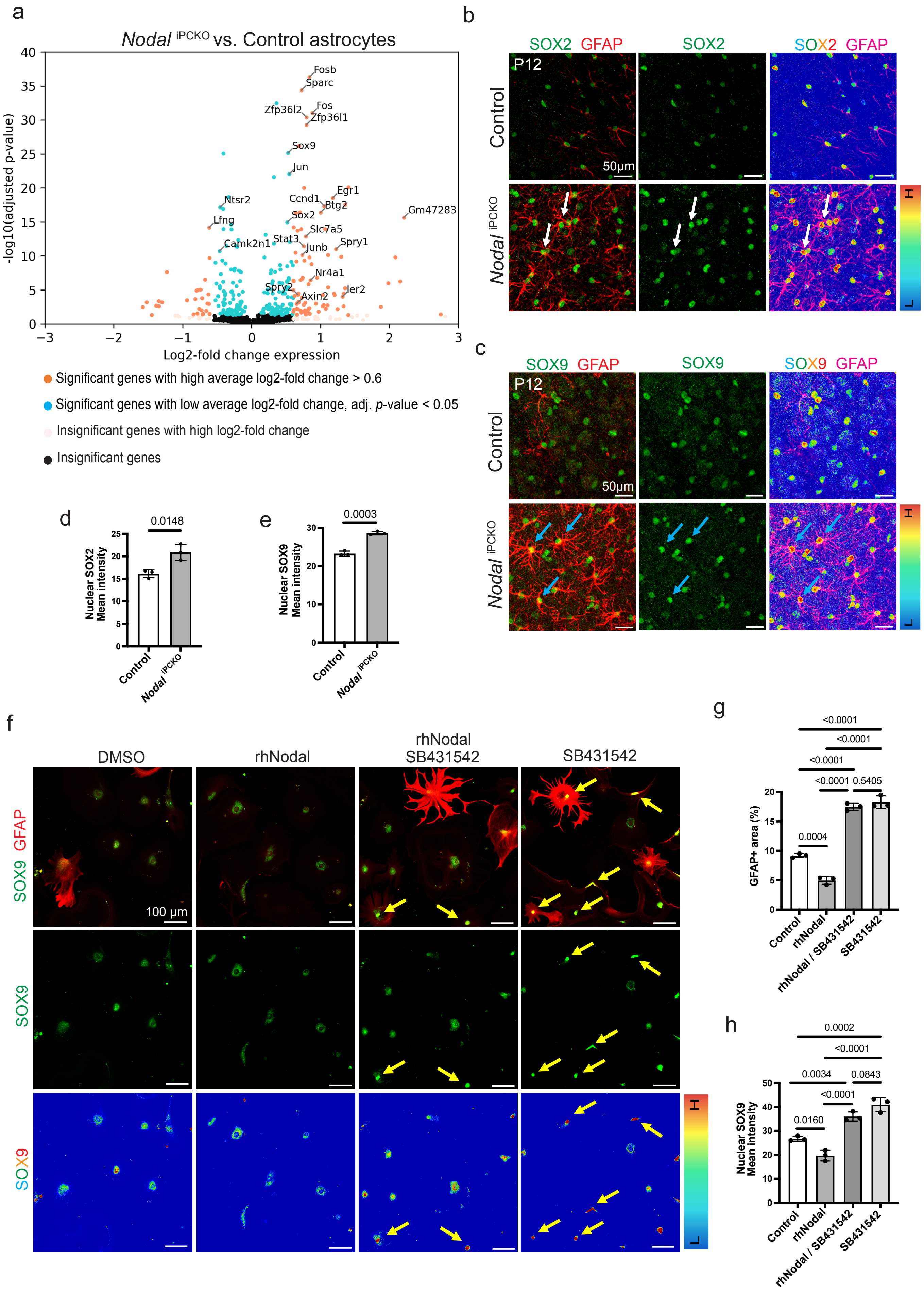
a
Nodal iPCKO vs. Control astrocytes
-log10(adjusted p-value)
Log2-fold change expression
Fosb
Sparc
Zfp36l2
Fos
Zfp36l1
Sox9
Jun
Egr1
Ccnd1
Btg2
Gm47283
Ntsr2
Sox2
Lfng
Slc7a5
Stat3
Camk2n1
Spry1
Junb
Nr4a1
Spry2
Ier2
Axin2
Significant genes with high average log2-fold change > 0.6
Significant genes with low average log2-fold change, adj. p-value < 0.05
Insignificant genes with high log2-fold change
Insignificant genes
b
SOX2 GFAP
SOX2
SOX2 GFAP
P12
Control
Nodal iPCKO
50μm
c
SOX9 GFAP
SOX9
SOX9 GFAP
P12
Control
Nodal iPCKO
50μm
d
Nuclear SOX2 Mean intensity
0.0148
Control
Nodal iPCKO
e
Nuclear SOX9 Mean intensity
0.0003
Control
Nodal iPCKO
f
DMSO
rhNodal
rhNodal SB431542
SB431542
SOX9 GFAP
SOX9
SOX9
100 μm
g
GFAP+ area (%)
<0.0001
<0.0001
<0.0001
<0.0001
0.5405
0.0004
Control
rhNodal
rhNodal / SB431542
SB431542
h
Nuclear SOX9 Mean intensity
0.0002
<0.0001
0.0034
0.0843
<0.0001
0.0160
Control
rhNodal
rhNodal / SB431542
SB431542

Supplementary Figure 14. Nodal-dependent expression of reactive astrocyte markers.

(a) Volcano plot showing differential gene expression in astrocytes from *Nodal* ^{iPCKO} and littermate control cortex. Average log2-fold change > 0.6, adjusted p-value < 0.05). Pseudobulk DE analysis uses two-sided Wald test + independent filtering as implemented by pyDESeq2.

(b, c) Confocal images of P12 *Nodal* ^{iPCKO} and littermate control brain cortex. Mutant GFAP+ (red) astrocytes show nuclear enrichment of Sox2 (green, white arrows) (**b**) as well as Sox9 (green, blue arrows) compared to control astrocytes (**c**). Heatmap; low intensity (blue) to high intensity (red) in **b** and **c**.

(d, e) Graphs comparing the nuclear enrichment of SOX2 **(e)** and SOX9 **(e)** in P12 *Nodal* ^{iPCKO} and littermate control brain cortex. Data represents mean ± s.e.m. (n=3 in **d, e**); P-value, unpaired two tailed student t-test.

(f) Expression of GFAP (red) and nuclear enrichment of Sox9 (green) in cultured mouse primary astrocytes treated with rhNodal and SB431542 inhibitor. Inhibition of TGFβ signaling increases nuclear SOX9 (yellow arrows). Heatmap; low intensity (blue) to high intensity (red).

(g, h) Graphs showing that Nodal signaling reduces the expression of GFAP **(f)** and nuclear enrichment of SOX9 **(g)** in cultured mouse primary astrocytes, whereas SB431542 inhibitor has the opposite effect. Data represents mean ± s.e.m. (n=3 in **g, h**); P-value, one-way ANOVA with Tukey's test in **g** and **h**.

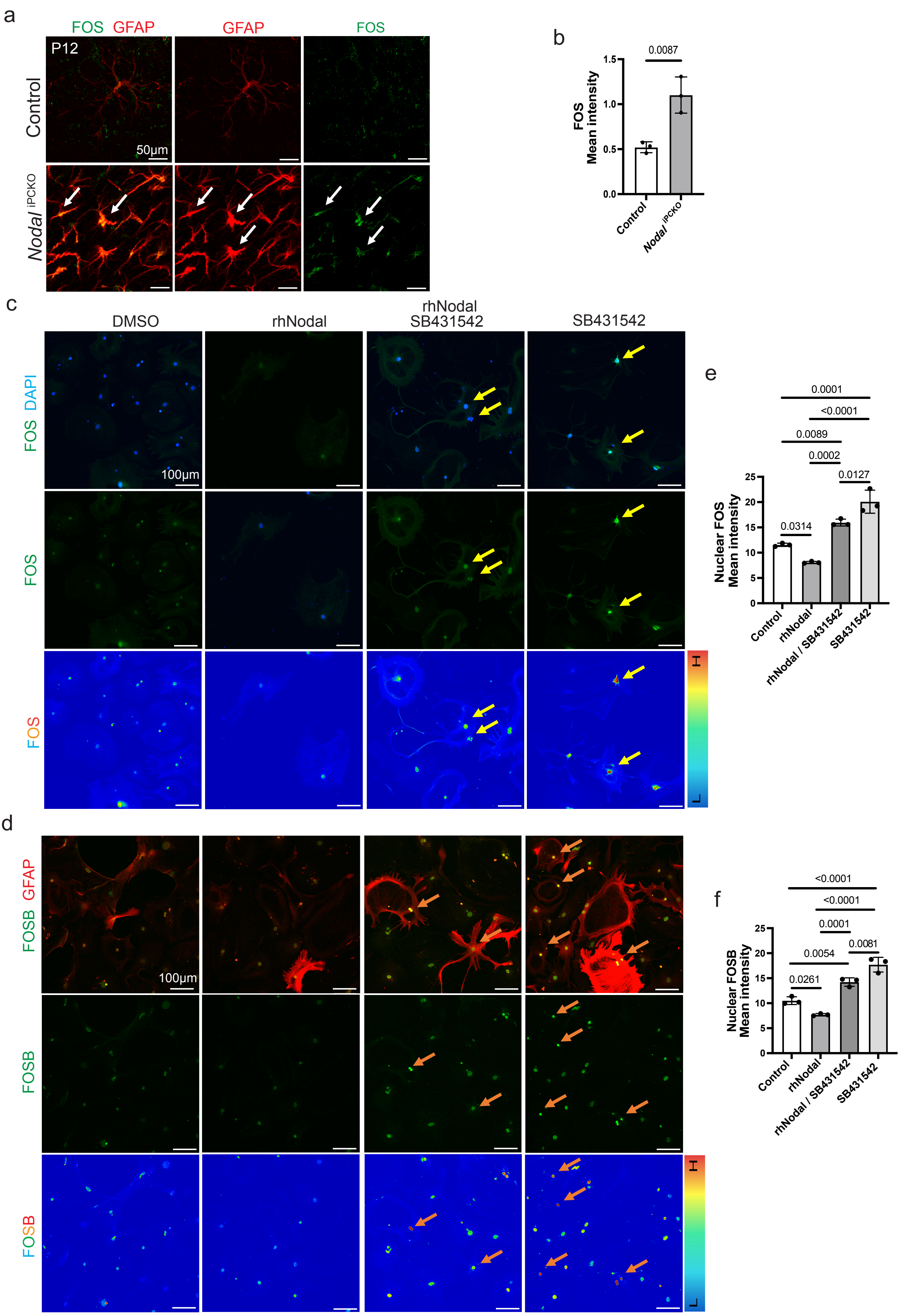

a
FOS GFAP
GFAP
FOS
P12
Control
50μm
Nodal iPCKO
b
0.0087
FOS Mean intensity
1.5
1.0
0.5
0.0
Control
Nodal iPCKO
c
DMSO
rhNodal
rhNodal SB431542
SB431542
FOS DAPI
100μm
FOS
FOS
H
L
e
0.0001
<0.0001
0.0089
0.0002
0.0127
0.0314
Nuclear FOS Mean intensity
25
20
15
10
5
0
Control
rhNodal
rhNodal / SB431542
SB431542
d
FOSB GFAP
100μm
FOSB
FOSB
H
L
f
<0.0001
<0.0001
0.0001
0.0081
0.0054
0.0261
Nuclear FOSB Mean intensity
25
20
15
10
5
0
Control
rhNodal
rhNodal / SB431542
SB431542

Supplementary Figure 15. TGFβ signaling controls levels of nuclear FOS and FOSB.

(a) Confocal images of brain cortex stained for FOS (green) and GFAP (red). White arrows indicate nuclear enrichment of FOS in *Nodal* ^{iPCKO} astrocytes.

(b) Graph showing the enhanced expression of FOS in *Nodal* ^{iPCKO} astrocytes compared to control. Data represents mean ± s.e.m. (n=3); P-value, unpaired two tailed student t-test.

(c, d) Inhibition of TGFβ signaling with SB431542 increases nuclear FOS (green, yellow arrows) (**c**) and FOSB (green, orange arrows) (**d**) in cultured mouse primary astrocytes. Heatmap; low intensity (blue) to high intensity (red) in **c**, **d**.

(e, f) Graphs showing that Nodal signaling reduces the nuclear enrichment of FOS **(e)** and FOSB in cultured mouse primary astrocytes, whereas SB431542 inhibitor has the opposite effect. Data represents mean ± s.e.m. (n=3 in **e, f**); P-value, one-way ANOVA with Tukey's test in **e, f**.

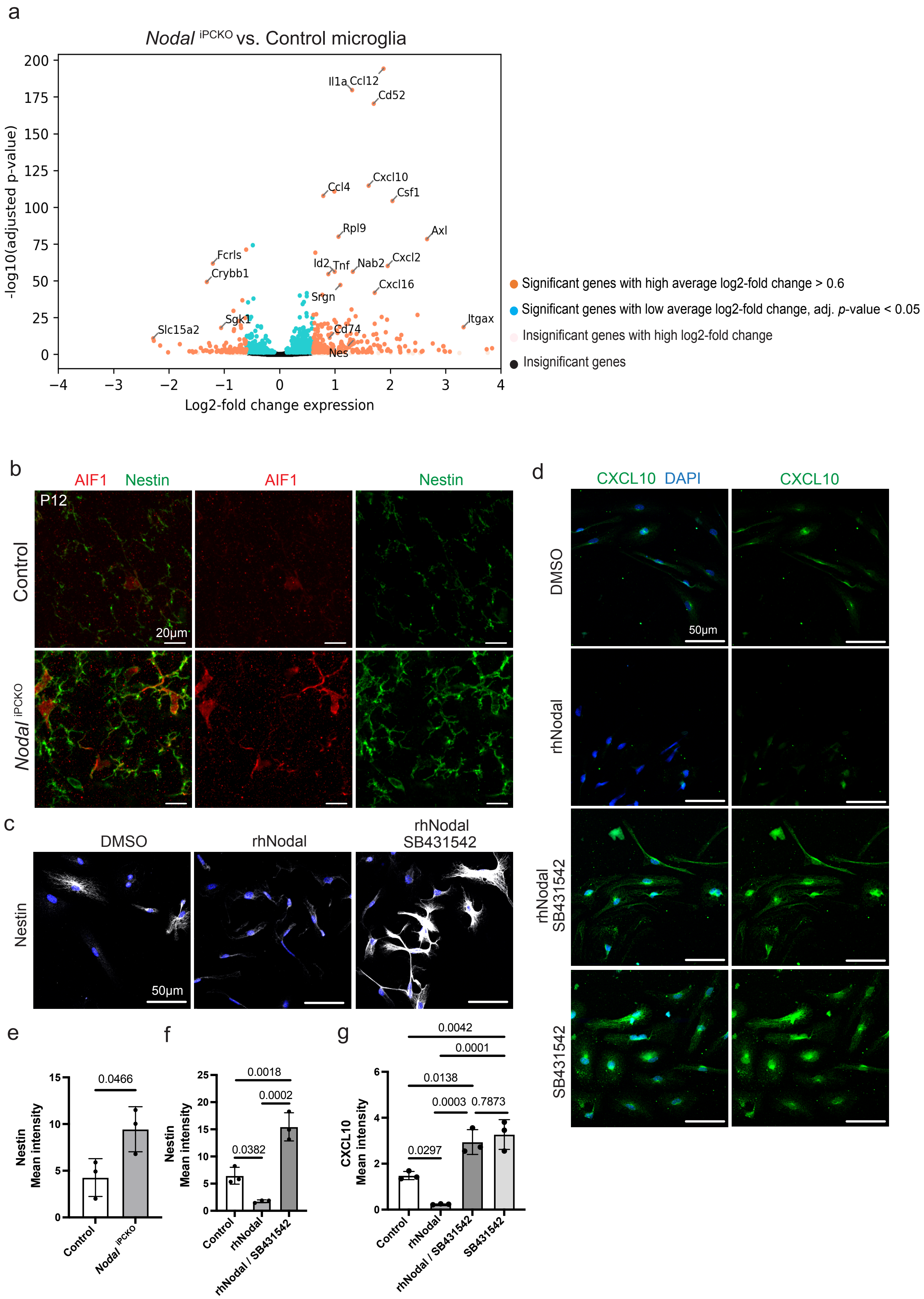
a
Nodal iPCKO vs. Control microglia
-log10(adjusted p-value)
Log2-fold change expression
Il1a
Ccl12
Cd52
Cxcl10
Ccl4
Csf1
Rpl9
Axl
Fcrls
Crybb1
Id2
Tnf
Nab2
Cxcl2
Cxcl16
Srgn
Sgk1
Slc15a2
Cd74
Itgax
Nes
Significant genes with high average log2-fold change > 0.6
Significant genes with low average log2-fold change, adj. p-value < 0.05
Insignificant genes with high log2-fold change
Insignificant genes
b
AIF1 Nestin
AIF1
Nestin
P12
Control
Nodal iPCKO
20µm
c
DMSO
rhNodal
rhNodal SB431542
Nestin
50µm
d
CXCL10 DAPI
CXCL10
DMSO
rhNodal
rhNodal SB431542
SB431542
50µm
e
Nestin Mean intensity
0.0466
Control
Nodal iPCKO
f
Nestin Mean intensity
0.0018
0.0002
0.0382
Control
rhNodal
rhNodal / SB431542
g
CXCL10 Mean intensity
0.0042
0.0001
0.0138
0.0003
0.7873
0.0297
Control
rhNodal
rhNodal / SB431542
SB431542

Supplementary Figure 16. Marker genes of activated microglia are negative regulated by Nodal signaling.

(a) Volcano plot showing the differential expression of genes in *Nodal* ^{iPCKO} microglia relative to control cells. Average log2-fold change > 0.6, adjusted p-value < 0.05). Pseudobulk DE analysis uses two-sided Wald test + independent filtering as implemented by pyDESeq2.

(b) Increased expression of Allograft inflammatory factor-1 (AIF1, red) and Nestin (green) in *Nodal*^{iPCKO} microglia compared to control.

(c, d) Confocal images showing Nestin **(c)** and CXCL10 **(d)** expression in primary microglia treated with Nodal and SB431542 inhibitor.

(e) Graph showing the increased expression of Nestin in *Nodal* ^{iPCKO} sections of brain cortex compared to control littermate. Data represents mean ± s.e.m. (n=3); P-value, unpaired two tailed student t-test.

(f, g) Graphs showing that Nodal signaling reduces the expression of Nestin **(f)** and CXCL10 **(g)** in cultured mouse primary microglia, whereas SB431542 inhibitor has the opposite effect. Data represents mean ± s.e.m. (n=3 in **f, g**); P-value, one-way ANOVA with Tukey's test in **f, g**.

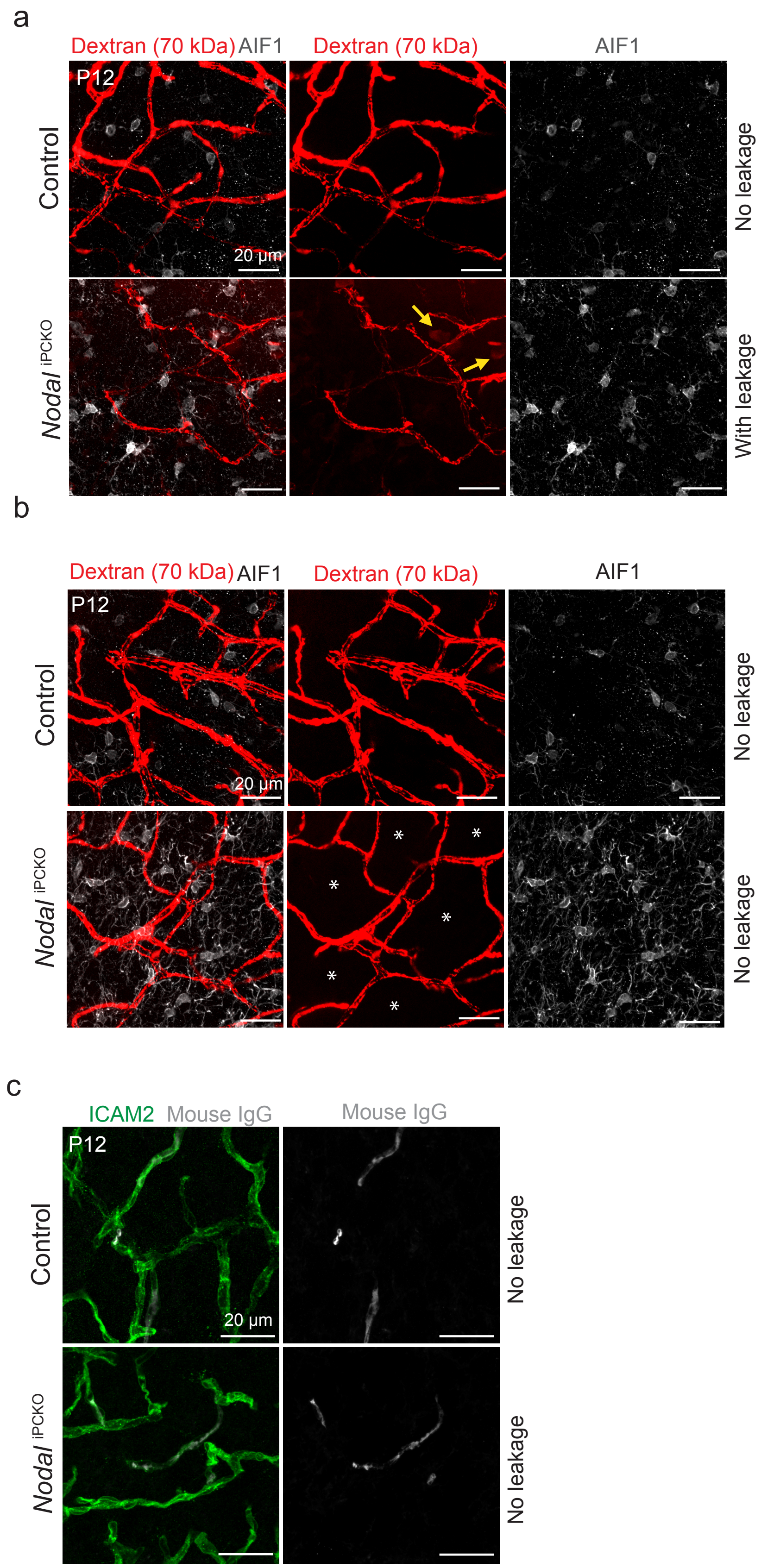
a
Dextran (70 kDa) AIF1
Dextran (70 kDa)
AIF1
P12
Control
20 µm
No leakage
Nodal iPCKO
With leakage
b
Dextran (70 kDa) AIF1
Dextran (70 kDa)
AIF1
P12
Control
20 µm
No leakage
Nodal iPCKO
No leakage
c
ICAM2 Mouse IgG
Mouse IgG
P12
Control
20 µm
No leakage
Nodal iPCKO
No leakage

Supplementary Figure 17. Microglia activation in the *Nodal* ^{iPCKO} brain cortex.

(a, b) Confocal images of P12 brain cortex stained for microglial marker AIF1 (grey) from *Nodal*^{iPCKO} and control littermates injected with Texas Red-dextran (70 kDa) (red), with local leakage (yellow arrows) **(a)**. Activated microglia can also be observed in areas of *Nodal* ^{iPCKO} cortex with no leakage (asterisks) **(b)**.

(c) Confocal images of brain cortex stained for ICAM2 (green) and mouse immunoglobulin G (IgG, white) showing region lack of extravasated IgG in P12 *Nodal*^{iPCKO} and control littermates (6/14 brains).

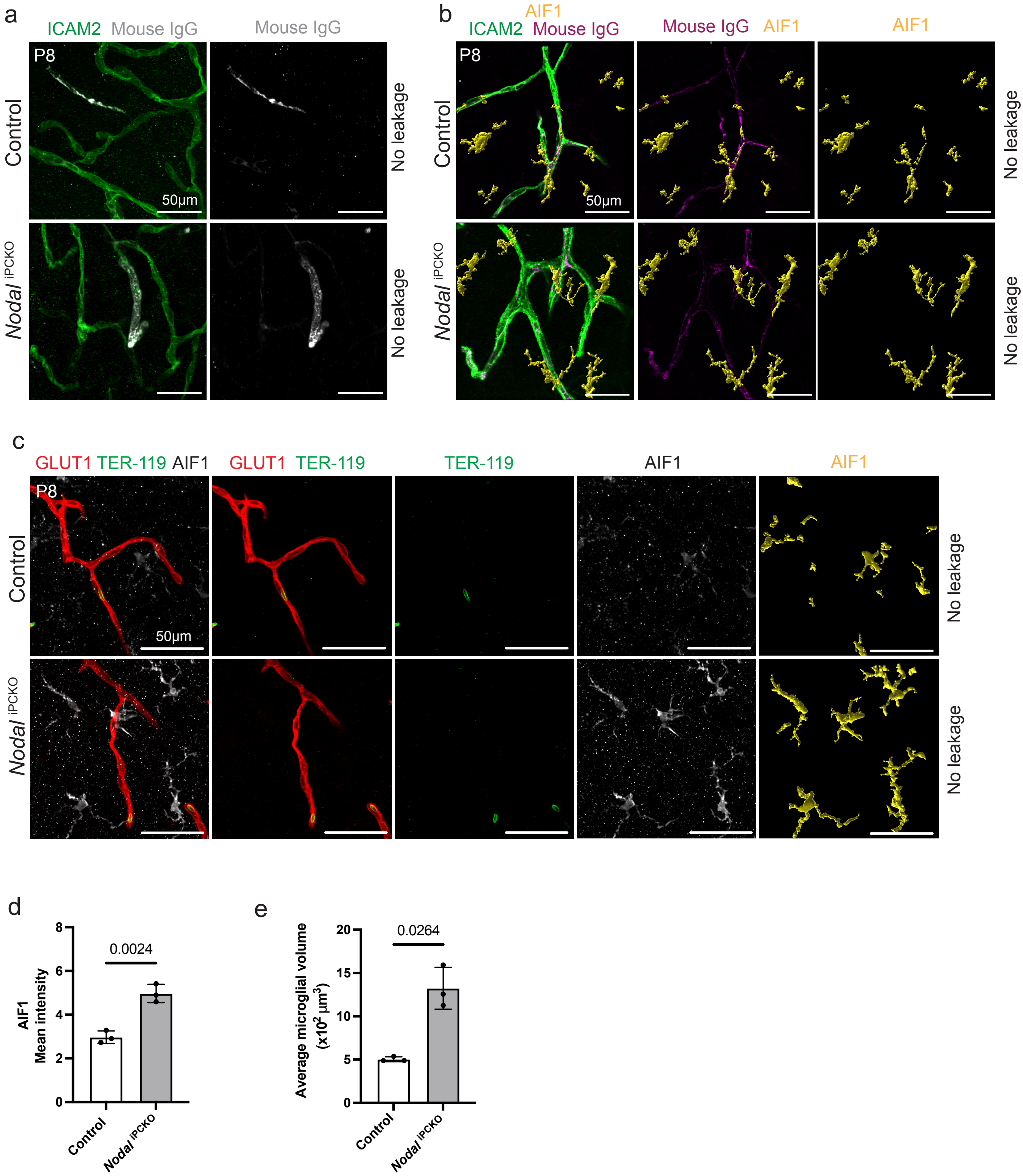

a
ICAM2 Mouse IgG
Mouse IgG
P8
Control
50μm
No leakage
Nodal iPCKO
No leakage
b
AIF1
ICAM2 Mouse IgG
Mouse IgG AIF1
AIF1
P8
Control
50μm
No leakage
Nodal iPCKO
No leakage
c
GLUT1 TER-119 AIF1
GLUT1 TER-119
TER-119
AIF1
AIF1
P8
Control
50μm
No leakage
Nodal iPCKO
No leakage
d
AIF1
Mean intensity
0.0024
Control
Nodal iPCKO
e
Average microglial volume
(x10² μm³)
0.0264
Control
Nodal iPCKO

Supplementary Figure 18. Microglia activation emerges early in *Nodal* ^{iPCKO} cortex.

(a, b) Confocal images of brain cortex stained for ICAM2 (green) and mouse IgG showing lack of extravasated IgG in P8 *Nodal*^{iPCKO} and control littermates (3/3 brains) **(a, b)**. Imaris surface-rendered image of AIF1+ microglia (yellow) showing that microglia volume is increased in P8 *Nodal*^{iPCKO} mutants **(b)**.

(c) Confocal images of GLUT1+ (red) capillaries in brain cortex. Red blood cells (Ter119, green) are confined to vessels in P8 *Nodal*^{iPCKO} and control littermates (3/3 brains), but increased microglia volume (white) can also be observed in P8 *Nodal*^{iPCKO} mutants.

(d, e) Quantitation of AIF1 expression **(d)** and average microglial volume **(e)** in the P8 *Nodal* ^{iPCKO} and littermate control brain cortex. Data represents mean ± s.e.m. (n=3); P-value, unpaired two tailed student t-test in **d** and unpaired two tailed Welch's test in **e**.
