# Supplementary material for: Pericytes are organ-specific regulators of tissue morphogenesis
Source: Nat Commun. 2026 May 12;17:4229. doi: 10.1038/s41467-026-71643-1 (PMC13168628; doi:10.1038/s41467-026-71643-1)
Supplement: Supplementary file 2 — Description of Additional Supplementary Files [file 41467_2026_71643_MOESM2_ESM.pdf]

Description of Additional Supplementary Files

File Name: Supplementary Data 1

Description: Full list of additional secreted factors that are significantly and uniquely expressed in either lung or brain.

File Name: Supplementary Data 2

Description: Full list of additional secreted factors that are significantly expressed in either the lung or the brain.

File Name: Supplementary Data 3

Description: Full list of genes that encode secreted factors and are differentially expressed in lung and brain pericytes.

File Name: Supplementary Data 4

Description: Full list of DEGs distinguishing lung and brain pericytes.
Pseudobulk DE analysis uses two-sided Wald test + independent filtering as implemented by pyDESeq2 in Supplementary Data 1-4.
